# Predictive ability of hypotension prediction index and machine learning methods in intraoperative hypotension: a systematic review and meta-analysis

**DOI:** 10.1186/s12967-024-05481-4

**Published:** 2024-08-05

**Authors:** Ida Mohammadi, Shahryar Rajai Firouzabadi, Melika Hosseinpour, Mohammadhosein Akhlaghpasand, Bardia Hajikarimloo, Roozbeh Tavanaei, Amirreza Izadi, Sam Zeraatian-Nejad, Foolad Eghbali

**Affiliations:** 1 Cardiovascular Surgery Research and Development Committee, Iran University of Medical Sciences (IUMS), Tehran, 14665-354 Iran; 2 Department of Surgery, Surgery Research Center, School of Medicine, Rasool-E Akram Hospital, Iran University of Medical Sciences, Tehran, Iran

**Keywords:** Intraoperative hypotension, Artificial intelligence, Machine learning, Deep learning, Anesthesia

## Abstract

**Introduction:**

Intraoperative Hypotension (IOH) poses a substantial risk during surgical procedures. The integration of Artificial Intelligence (AI) in predicting IOH holds promise for enhancing detection capabilities, providing an opportunity to improve patient outcomes. This systematic review and meta analysis explores the intersection of AI and IOH prediction, addressing the crucial need for effective monitoring in surgical settings.

**Method:**

A search of Pubmed, Scopus, Web of Science, and Embase was conducted. Screening involved two-phase assessments by independent reviewers, ensuring adherence to predefined PICOS criteria. Included studies focused on AI models predicting IOH in any type of surgery. Due to the high number of studies evaluating the hypotension prediction index (HPI), we conducted two sets of meta-analyses: one involving the HPI studies and one including non-HPI studies. In the HPI studies the following outcomes were analyzed: cumulative duration of IOH per patient, time weighted average of mean arterial pressure < 65 (TWA-MAP < 65), area under the threshold of mean arterial pressure (AUT-MAP), and area under the receiver operating characteristics curve (AUROC). In the non-HPI studies, we examined the pooled AUROC of all AI models other than HPI.

**Results:**

43 studies were included in this review. Studies showed significant reduction in IOH duration, TWA-MAP < 65 mmHg, and AUT-MAP < 65 mmHg in groups where HPI was used. AUROC for HPI algorithms demonstrated strong predictive performance (AUROC = 0.89, 95CI). Non-HPI models had a pooled AUROC of 0.79 (95CI: 0.74, 0.83).

**Conclusion:**

HPI demonstrated excellent ability to predict hypotensive episodes and hence reduce the duration of hypotension. Other AI models, particularly those based on deep learning methods, also indicated a great ability to predict IOH, while their capacity to reduce IOH-related indices such as duration remains unclear.

**Supplementary Information:**

The online version contains supplementary material available at 10.1186/s12967-024-05481-4.

## Introduction

Every year more than 300 million surgeries are conducted worldwide [1], resulting in a significant number of patients experiencing possible intraoperative complications. One of the most common complications associated with both cardiac and non-cardiac surgeries is intraoperative hypotension (IOH) [2]. In non-cardiac surgeries, IOH has been associated with myocardial injury [3], acute kidney injury [4], and death [5]. Additionally, in cardiac surgeries hypotension during cardiopulmonary bypass (CPB) has been associated with a significant risk of ischemic stroke, with the risk increasing as more time is spent in a hypotensive state. As a consequence, it is imperative that the duration of IOH be minimized during surgeries. The current model of managing IOH is mostly reactive, and the treatment often occurs with delay [6]. However, the perilous ramifications of IOH have recently pushed researchers towards more proactive approaches to its treatment.

Even though efforts have been made to identify the epidemiological factors predisposing patients to IOH in order to estimate its risk during surgeries [7, 8], they are not helpful in reducing IOH in clinical settings [9]. However, AI models have demonstrated high efficiency in predicting IOH in real-time and providing the clinician with enough time to act before the onset of a hypotensive episode. Furthermore, AI model have proven useful in predicting and preventing hypotension-induced complications, including sepsis [10] and acute kidney injury [11]. These real-time IOH prediction models have been proven so valuable that one logistic regression (LR) model in particular_ the Hypotension Prediction Index (HPI) [12]_ is now commercially available for use, and has prompted many clinical trials and validation studies testing its efficiency in predicting IOH.

As studies assessing the efficiency of AI models including HPI have been accumulating in the literature, we set out to compile the existing evidence regarding the efficacy of such models and HPI by area under the receiver operating curve (AUROC). This review aims to examine the current state of AI research in IOH prediction. Further, it aims to quantify and compare the efficiency and predictive ability of both HPI and non-HPI models.

## Methods

This systematic review and meta-analysis was conducted in accordance with PRISMA (Preferred Reporting Items for Systematic Reviews and Meta-Analyses) guidelines [13] in search of papers developing or validating artificial intelligence methods for the prediction of intraoperative hypotension. The protocol for this review was prospectively registered on PROSPERO (CRD42024504636). Due to the high number of studies evaluating the hypotension prediction index (HPI), those studies were extracted and analyzed separately.

### Search strategy

An online database search of Pubmed, Scopus, Embase, and Web of Science was conducted on December 9th, 2023 with MeSH terms and keywords synonymous with “artificial intelligence” and “intraoperative hypotension”. No publication date or language limitations were defined. A manual citation search of the included studies was also performed after finalization of the screening process.

### Eligibility criteria

For the purposes of our study IOH was defined as hypotension occurring after the induction of anesthesia, regardless of the specific blood pressure cut-off that was utilized (e.g. MAP < 65 mm Hg, or MAP < 55 mm Hg) or timeframe during which IOH was detected by different studies (e.g. from tracheal intubation to incision, after incision, etc.).

Studies were included if they complied with the following PICOS:

Population: patients undergoing cardiac or non-cardiac surgeries with intraoperative blood pressure monitoring.

Intervention: predictive models utilizing artificial intelligence which are either being developed or being validated.

Comparator: standard intraoperative care or non-AI models, if applicable.

Outcomes: area under the receiver operating characteristics curve for prediction of intraoperative hypotension as defined by the study using AI models that have been developed or are being validated. Duration of hypotension as defined by the study or time-weighted average of hypotension (TWA-MAP < 65 mmHg) or area under the threshold for hypotension (AUT-MAP < 65 mmHg) for AI models under validation.

Study design: AI development papers or controlled studies validating AI models.

Papers were excluded if they did not include human participants, only used AI models for feature selection, or if they were conference abstracts.

### Study selection

Studies were screened by two independent reviewers in two phases. An initial title abstract screening followed by full-text retrieval and evaluation. Discrepancies between the two were resolved by an independent third reviewer through discussion.

### Data extraction

The following data were extracted into one of two pre-constructed Excel spreadsheets by two independent reviewers under the supervision of a third reviewer. The first spreadsheet contained the following data regarding studies not utilizing the hypotensive prediction index: author, year of publication, country, type of surgery (cardiac or non-cardiac), type of the source of dataset, population size, population age, population gender, predicted outcome, number of variables used in the model, mode of validation, best-performing algorithm, number of hypotensive events, number of hypotensive patients, and area under the receiver operating curve (AUROC).

The second spreadsheet included the following data regarding studies employing the hypotension prediction index: author, year of publication, country, type of study, type of surgery, type of comparator, group size, age and gender of the groups, number of intraoperative hypotensive events, number of hypotensive patients, duration of intraoperative hypotension, time-weighted average of hypotension (MAP < 65 mmHg), area under the threshold of MAP < 65 mmHg (mmHg*min), and AUROC for the hypotensive prediction index.

### Outcomes

The following were chosen as the main outcomes of this review: AUROC for HPI and non-HPI studies and TWA-MAP < 65, duration of intraoperative hypotension, and AUT-MAP < 65 for HPI studies only.

### Quality assessment

Quality assessment of non-HPI studies was conducted using the PROBAST tool [14], while quality assessment of studies utilizing HPI was done using the Jadad scale [15] or Newcastle-Ottowa scale [16] for controlled trials and observational studies respectively.

### Statistical analysis

Meta-analysis of the duration of hypotension, TWA-MAP < 65, and AUT-MAP < 65 of the HPI studies was conducted using the Comprehensive Meta-Analysis software (CMA, version 3, NJ, USA) utilizing a random effects model with standardized mean difference (SMD) and its corresponding 95% confidence interval (CI) as the effect size. Means and standard deviations were the only accepted data entry form and medians with interquartile ranges were converted to means and standard deviations using methods outlined by Wan et al. [17] and Luo et al. [18].

Meta-analysis of the AUROC for both the HPI and non-HPI studies was done using Stata version 18 (StataCorp. 2023. Stata Statistical Software; College Station, TX, USA), employing a random effects model with the restricted maximum-likelihood method. AUROC of the HPI studies was sub-group meta-analyzed by the time before prediction of intraoperative hypotension, while the AUROC of the non-HPI studies was sub-group analyzed by algorithm type, and the definition of IOH used to construct the models. In addition, subgroup analyses were conduceted for all of the primary outcomes in both types of studies according to the quality of the studies.

Heterogeneity was assessed using the I^2^ statistic, with an I^2^ > 50% signifying substantial heterogeneity [19]. A *p*-value of < 0.05 was considered statistically significant. Sensitivity analysis was conducted using the leave-one-out method and publication bias was assessed using Egger’s regression test (*p*-value < 0.05) and funnel plot symmetry if at least 10 studies were included in the meta-analysis.

## Results

**Fig. 1:**
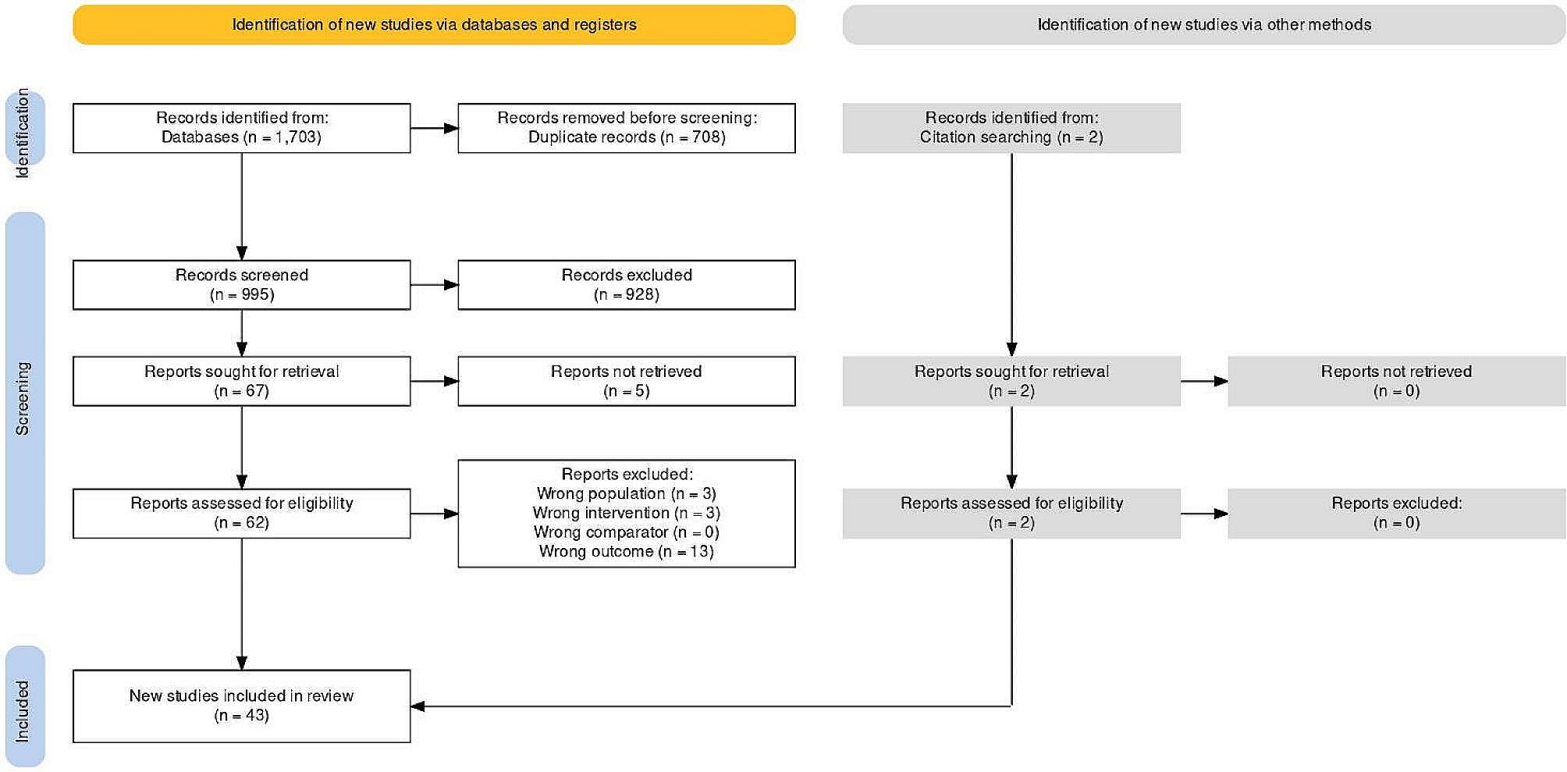
PRISMA flowchart made using the Shiny app by Haddaway et al. [20]

The initial search yielded 1705 records, with 997 studies eligible for screening after duplicate removal. A total of 67 studies were then chosen for full-text evaluation and 43 of them were included in our review. 22 of the included studies were HPI studies, while 21 studies developed non-HPI models to predict IOH (Fig. 1).

### HPI studies

The 22 HPI studies were published between 2018 and 2023, 13 of which were cohort studies [21–33], 8 were controlled trials [34–41] and one was the model development study [12]. Almost all of the HPI studies investigated the algorithm’s efficacy in non-cardiac surgeries, with the exception of Davies et al. [29] who did not exclude cardiac surgeries and Shin et al. [32] who only included cardiac surgeries. The number of participants in each study ranged between 20 [24] to 874 [12] and the mean/median age of participants ranged between 36 [27] to around 70 [35] years old, with most studies including participants with a mean/median age between 60 and 70 years old. 4 of the HPI studies investigated obstetric surgeries and only included females [26–28, 34] but the rest of the studies included males and females in relatively equal proportions, except for two studies [24, 32]. (Table 1; Additional file 1: Table S1)

**Table 1:** Characteristics of HPI studies

Study characteristics					NO of patients		Age		Gender (male)	
First author and year	Country	Type of study	Type of surgery	Type of Comparator	Sample	Control	Sample	Control	Sample	Control
**Hatib**,** 2018** [12]	USA	AI development	Non-cardiac	NA	Train = 293 Test = 350 External validation = 204	NA	Train = 61.4 Test = 61.9 External validation = 57	NA	Train = 62.4% Test = 62.8% External validation = 49%	NA
**Frassanito**,** 2022** [26]	Italy	Cohort	Non-cardiac (Major Gynecologic Oncologic)	NA	28	NA	50	NA	0	NA
**Frassanito**,** 2023** [28]	Italy	Cohort	Non-cardiac (Major Gynecologic Oncologic)	Pre-defined Goal Directed Therapy	22	22	NP	NP	0	0
**Frassanito**,** 2022** [27]	Italy	Cohort	Non-cardiac (Cesarean delivery)	NA	48	0	36	NA	0	NA
**Davies**,** 2020** [29]	UK	Cohort	Both	NA	255	0	68	NA	69.41	NA
**Grundmann**,** 2021** [25]	Germany	Cohort	Non-cardiac (elective major abdominal)	FloTrac	50	50	Median of 66	Median of 66.5	58	56
**Kouz**,** 2023** [21]	France, Germany, Italy, Spain, and United Kingdom	Cohort	Noncardiac (elective major general)	NA	702	NA	64	NA	52%	NA
**Maheshwari**,** 2021** [22]	USA	Cohort	Non-cardiac	NA	305	Na	60	NA	NP	NP
**Runge**,** 2023** [31]	Germany	Cohort	Non-cardiac	In house protocol	136	136	Median of 68.0	Median of 67	61.00%	62.50%
**Schenk**,** 2021** [30]	the Netherlands	Cohort	Non-cardiac	In house protocol	28	26	Median of 69	Median of 62	67.90%	42.30%
**Shin**,** 2021** [32]	USA	Cohort	Cardiac	NA	36	NA	63	NA	75.67%	NA
**Solares**,** 2023** [33]	Spain	Cohort	Non-cardiac	In house protocol	52	52	Median of 59	Median of 69	61.50%	61.50%
**Wijnberge**,** 2021** [23]	the Netherlands	Cohort	Non-cardiac	NA	507	NA	54.7	NA	45.00%	NA
**Yang**,** 2023** [24]	South Korea	Cohort	Non-cardiac (Liver transplant)	NA	20	NA	58.5	NA	75%	NA
**Frassanito**,** 2023** [34]	Italy	RCT	Non-cardiac (Major Gynecologic Oncologic)	Pre-defined Goal Directed Therapy	30	30	57.50	58.64	0	0
**Murabito**,** 2022** [35]	Italy	RCT	Non-cardiac (elective laparotomic major general)	In house protocol	20	20	median of 69	Median of 70.5	50	60
**Maheshwari**,** 2020** [36]	USA	RCT	Noncardiac	NP	105	108	67	66	53%	57%
**Schneck**,** 2019** [37]	Germany	RCT	Non-cardiac	NP	25	24	Median of 66	Median of 60	48%	54%
**Šribar**,** 2023** [38]	Croatia	RCT	Non-cardiac (thoracic)	Conventional pulse contour analysis	17	17	Median of 65	Median of 69	41.17%	52.94%
**Tsoumpa**,** 2021** [39]	Greece	RCT	Non-cardiac	Conventional treatment with invasive blood pressure monitoring	49	50	Median of 66	Median of 70	53%	58%
**Wijnberge**,** 2020** [40]	the Netherlands	RCT	Non-cardiac	In house protocol	31	29	Median of 68.0	Median of 62	68%	45%
**Yoshikawa**,** 2023** [41]	Japan	RCT	Non-cardiac	FloTrac	30	30	68	67	40%	47%

*Abbreviations*:

*NP: not provided/ NA: not applicable*

#### Duration of IOH

**Fig. 2:**
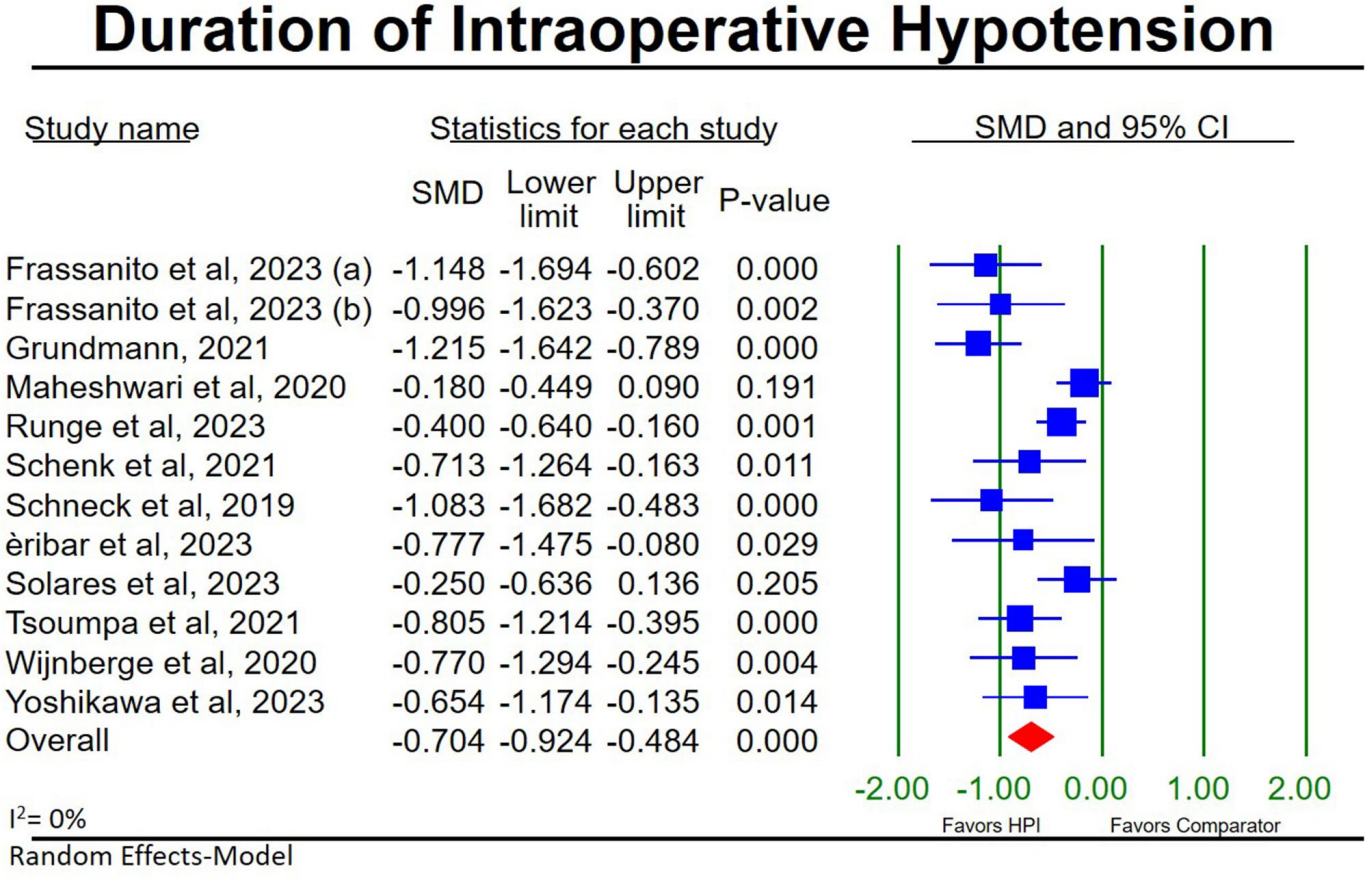
Forest plot for the meta-analysis of hypotension prediction index studies comparing the duration of intraoperative hypotension between hypotension prediction index guided participants and participants receiving standard in-house protocols. **a** [33]. **b** [27]

12 HPI studies [23, 26, 28, 29, 31, 32, 34–39] reported the duration of IOH for 575 HPI-guided participants compared to 574 participants in standard in-house protocols, the pooled results of which shows a significant reduction in the duration of IOH in the HPI guided participants (SMD, (95CI), *p*-value; -0.70, (-0.92, -0.48), < 0.001; Fig. 2) with low heterogeneity (I^2^ = 0%). Sensitivity analysis shows our findings to be stable (Additional file 2: Figure S1) yet significant publication bias is present (Egger’s test *p*-value = 0.007; Additional file 1: Figure S2).

#### TWA-MAP < 65 mmHg

11 HPI studies [25, 28, 30, 31, 33, 34, 36, 38–41] evaluated the TWA-MAP < 65 mmHg in 550 HPI-guided individuals compared to 550 individuals receiving standard in-house protocols. Our meta-analysis of these studies shows a significant reduction in the TWA-MAP < 65 mmHg in the HPI-guided groups (SMD, (95CI), *p*-value; -0.63, (-0.85, -0.42), < 0.001; Additional file 1: Fig. 3) with low heterogeneity (I^2^ = 0%). Sensitivity analysis shows our findings to be stable (Additional file 1: Figure S3), however, significant publication bias is present (Egger’s test *p*-value = 0.03; Additional file 2: Figure S4).

**Fig. 3:**
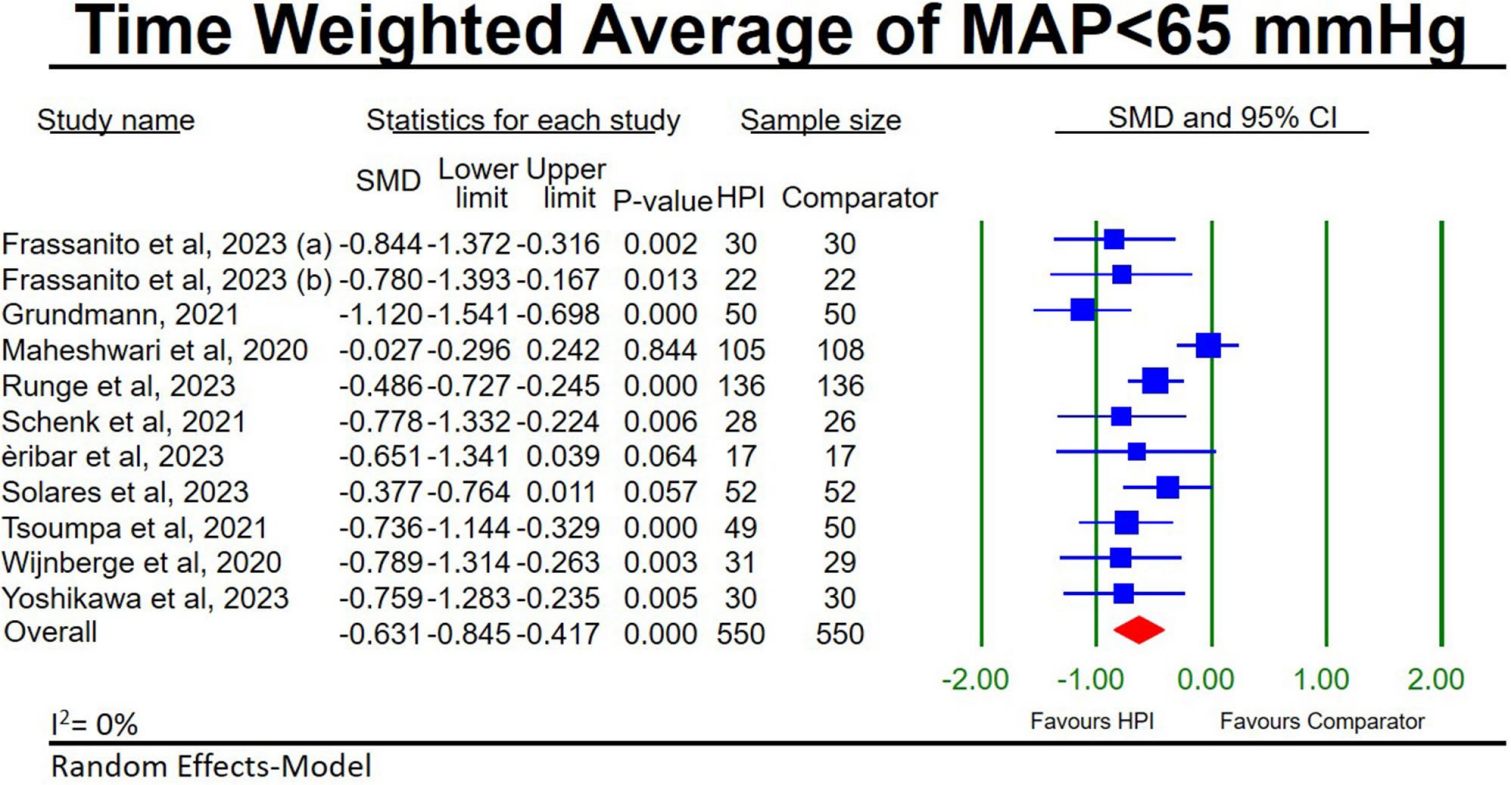
Forest plot for the meta-analysis of hypotension prediction index studies comparing the time-weighted average of hypotension (MAP < 65 mmHg) between hypotension prediction index guided participants and participants receiving standard in-house protocols. **a** [33]. **b** [27]

#### AUT-MAP < 65 mmHg

**Fig. 4:**
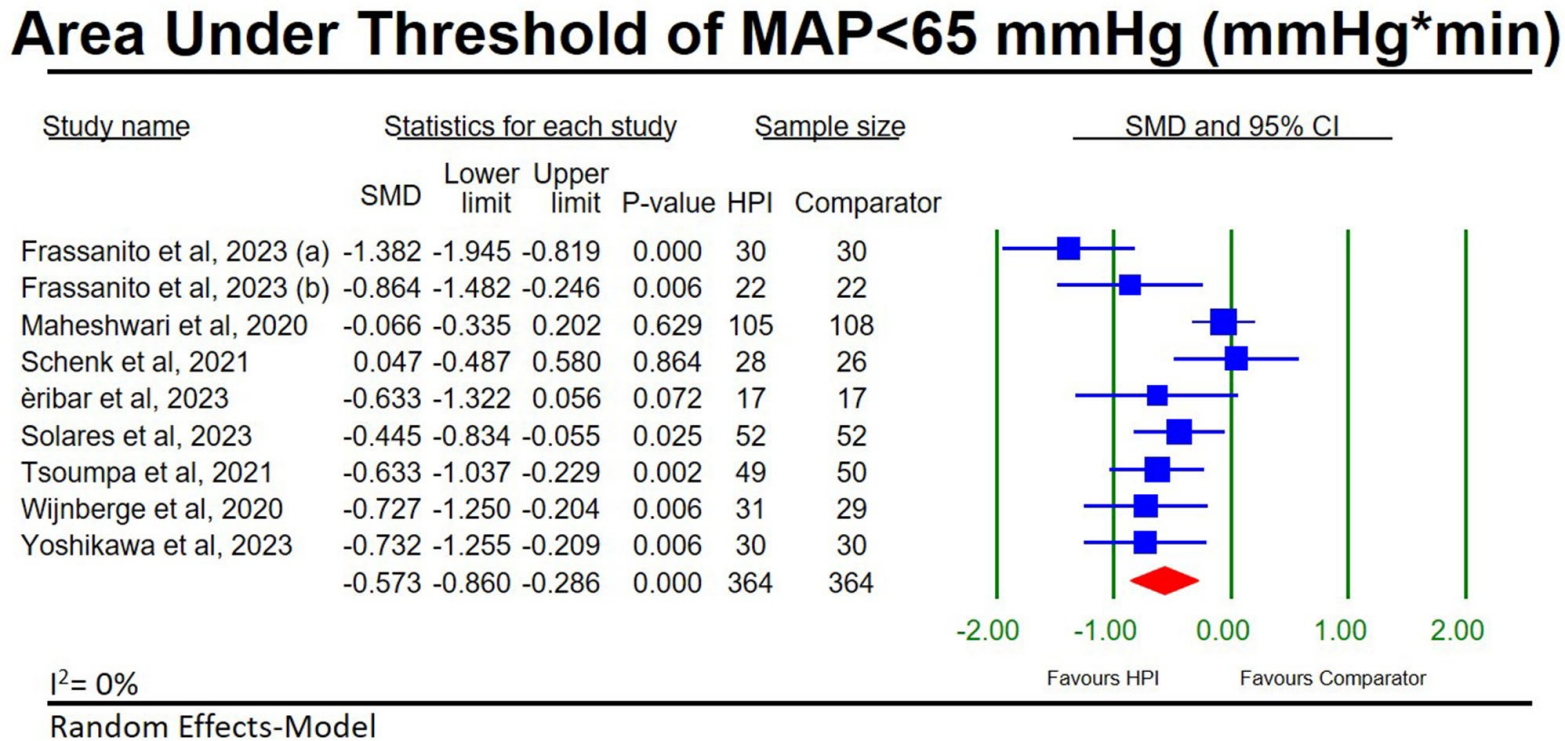
Forest plot for the meta-analysis of hypotension prediction index studies comparing the are under the threshold for hypotension (MAP < 65 mmHg) between hypotension prediction index guided participants and participants receiving standard in-house protocols. **a** [33]. **b** [27]

9 HPI studies [28, 30, 33, 34, 36, 38–41] investigated changes in AUT-MAP < 65 mmHg in 364 HPI-guided participants compared to 364 participants receiving standard in-house protocols. After pooling the studies together, we found a significant reduction in AUT-MAP < 65 mmHg in the HPI-guided participants (SMD, (95CI), *p*-value; -0.57, (-0.86, -0.29), < 0.001; Fig. 4) with low heterogeneity (I^2^ = 0%). Sensitivity analysis shows our results to be stable (Additional file 2: Figure S5) and publication bias was not assessed due to less than 10 studies being included in the meta-analysis.

#### AUROC

**Fig. 5:**
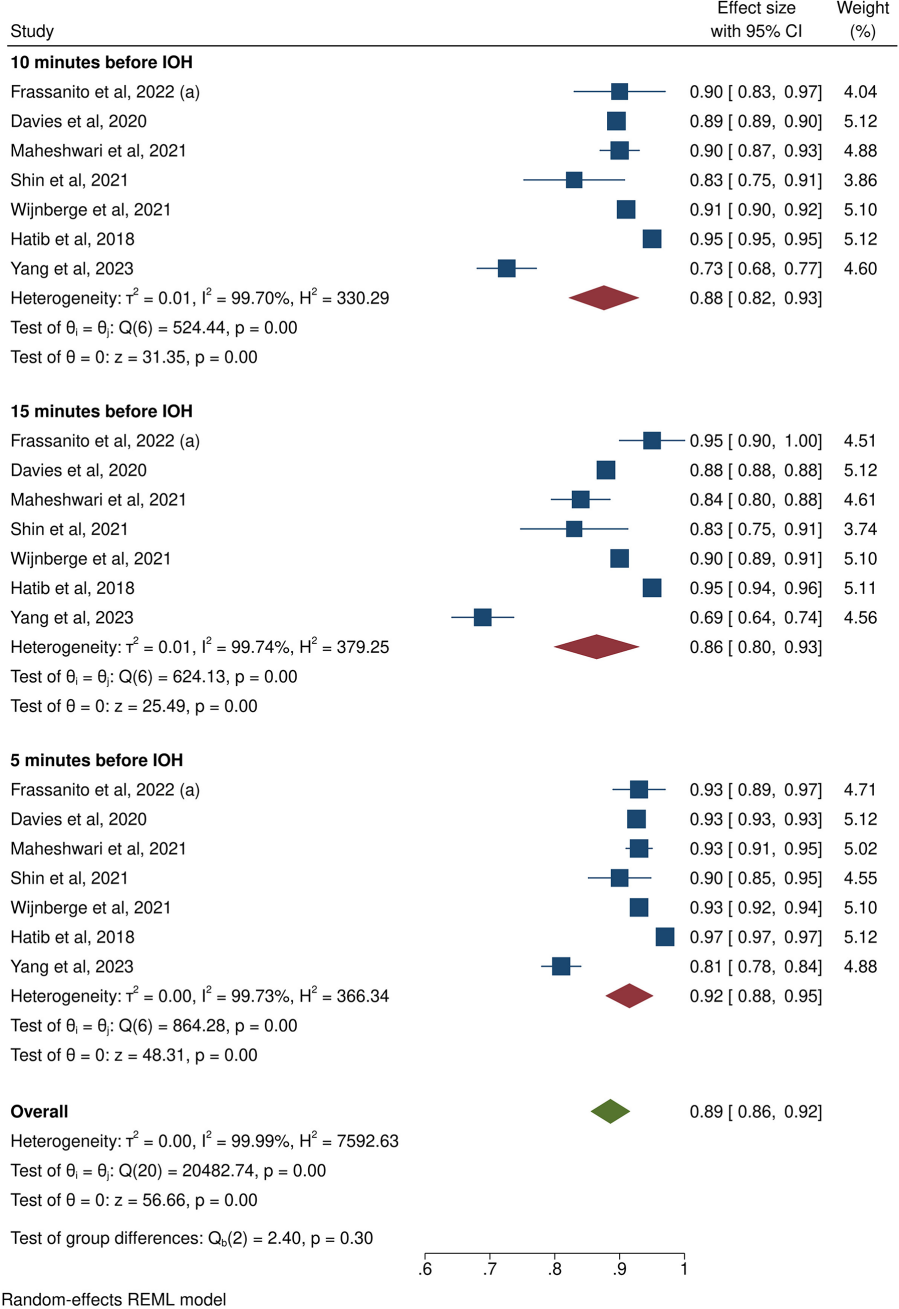
Meta-analysis of the AUROC of the hypotension prediction index at 5, 10, and 15 minutes before the occurrence of intraoperative hypotension. The overall AUROC of the hypotension prediction index for predicting intraoperative hypotension is presented at the bottom of the figure. **a** [25]

7 of the HPI studies assessed the performance of this algorithm in the prediction of IOH 5 to 15 min before the event [12, 22–24, 26, 29, 32]. After pooling the studies, we found the HPI algorithm has a considerable AUROC of 0.89 (95CI: 0.88, 0.92) between 5 and 15 min prior to the event. When stratified by time to event prediction, the algorithm performs insignificantly better 5 min before the event (AUROC: 0.92; 95CI: 0.88–0.95) in comparison to 10 min (AUROC: 0.88; 95CI: 0.82–0.93) and 15 min prior to IOH (AUROC: 0.86; 95CI: 0.80–0.93; Fig. 5). Significantly high heterogeneity is present in this meta-analysis (I^2^ = 99%) and inside each subgroup (I^2^ = 99%). Sensitivity analysis shows our findings to be stable (data not shown) and publication bias was not assessed due to less than 10 studies included in the meta-analysis.

### Non-HPI studies

A total of 21 studies developed original AI models to predict IOH using a wide host of algorithms, including random forest (RF) utilized in 7 studies [42–48], logistic regression (LR) used in 7 studies [44, 45, 49–53], convolutional neural networks (CNN) utilized in 5 studies [9, 46, 49, 54, 55], regular neural networks used in 4 studies [45, 56–58], gradient boosting machine (GBM) used in 4 studies [43, 45, 46, 53], deep neural networks (DNN) used in 4 studies [46, 52, 59, 60], artificial neural networks (ANN) utilized in 3 studies [44, 50, 51], and naive Bayesian used in 2 studies [44, 45]. Other algorithms were employed in one study only and include support vector machine [45], k-nearest neighbor [45], Linear Discriminant Analysis [45], recurrent neural networks (RNN) [49], Gaussian Process [61], Bayesian Neural Network [61], and Bayesian Generalized Linear Model [61]. These algorithms used between 4 [9] and 130 [43] features. (Additional file 1: Table S2)

The non-HPI studies were published between 2008 [51] and 2024 [61] and mostly based their models on datasets obtained from local electronic medical records (EMR) [9, 42–53, 55, 56, 59, 61], with only 4 studies utilizing a validated registry [54, 57, 58, 60]. The mean age of their datasets ranged from 31 [56] to 62 [48] years old and the mean age of most datasets hovered around 50 years old. Excluding the works by Dong et al. [61] and Feld et al. [53], which only used females or included mostly males respectively, other non-HPI studies used a gender-equal dataset (Table 2).

**Table 2:** Characteristics of the non-HPI AI development studies

First author and year	Country	Type of surgery	Type of source	NO. of patients	Age (mean)	Gender (male%)	Definition of IOH		Algorithm/ model	NO. of features	Mode of validation	Best model performance
							Cut-off	Time-frame				
Choe, 2021 [49]	South Korea	Non-cardiac	Local EMR	18,813 in total 13,178 in Train 5635 in validation	58.5	49.27	MAP < 65	Throughout surgery	STEP-OP (CNN + RNN) RNN CNN LR GP	12	Training/ validation	STEP-OP (CNN + RNN)
Dong, 2024 [61]	USA	Non-cardiac (hysterectomy)	Local EMR	562	52.55	0	NP	NP	BGLM BNN BNNMR	77	K-fold validation (K = 5)	Undecided
Gratz, 2020 [56]	USA	Non-cardiac (abdominal and obstetric surgeries)	Local EMR	49	31.7	NP	SBP < 90	Post-induction	NN single-feature discrimination	NP	Training/ validation / testing	NN
Greenbaum, 2020 [42]	South Korea	Non-cardiac (general surgery)	Local EMR	2084	NP	NP	A decrease in MAP of more than 15% from the prior MAP and a MAP lower than 65 mmHg	The first and last 30 min of each case were excluded to reduce confounding caused by induction and extubation	RF	NP	Training/ testing	NA
Inada, 2021 [43]	Japan	Not specified	Local EMR	1053	NP	47	SBP < 80 mmHg	Throughout surgery	RF GBM XGBoost LGBM	130	K-fold validation (K = 10)	RF
Jo, 2022 [54]	South Korea	Non-cardiac	Registry	14,140	58.8	50.5	MAP < 65	NP	CNN based on ECG, EEG and ABP	NP	Training/ validation/ testing	CNN (Using ABP + EEG data)
Kang, 2020 (44)	Republic of Korea	Non-cardiac (laparoscopic cholecystectomy)	Local EMR	222	53	46.90%	SBP < 90 mmHg or MAP < 65 mmHg	From tracheal intubation to incision	NB LR RF ANN	89	K-fold validation (K = 4)	RF
Kendale, 2018 [45]	USA	Both	Local EMR	13,323 Train: 9326 Test: 3997	50	44%	MAP < 55	Post-induction	SVM NB KNN LDA GBM RF LR NN	61	K-fold validation (K = 10)	GBM
Lee, 2020 [46]	South Korea	Non-cardiac (laparoscopic cholecystectomy)	Local EMR	282	54.7	47.50%	SBP < 90 mmHg or a mean blood pressure (MBP) < 65 mmHg	After tracheal intubation	RF XGBoost DNN CNN	27	K-fold validation (K = 10)	random forest
Lee, 2021 [9]	South Korea,	Non-cardiac	Registry	3301 Train:1980 Validation:330 Test:991	NP	NP	MAP < 65	NP	CNN	NP	Training/ validation/ testing	NA
Lee, 2022 [47]	South Korea	Non-cardiac (laparoscopic cholecystectomy)	Local EMR	888	No IOH: 58.75 IOH: 64.23	No Hypotension: 52.4% Hypotension: 56.2%	MAP < 65	NP	RF	36	K-fold validation (K = 5)	NA
Li, 2021 [48]	China	Cardiac surgery	Local EMR	3030 Train:2121 Test:909	Train:63 Test:62	67.2%	SBP < 90 or MAP < 65	Post-induction	RF	31	K-fold validation (K = 10)	NA
Lin, 2011 [50]	Taiwan	Both	Local EMR	1371 Train:1017 Validation:294	Train: 46.5 Validation: 46.4	Train: 36.8 Validation: 36.4	1. SBP less than 90 mm Hg or 2. SBP decrease more than 30%	Post-induction before the beginning of surgery	ANN LR	21	K-fold validation (K = 10) External validation	ANN
Lin, 2008 [51]	Taiwan	Non-cardiac (under spinal anesthesia)	Local EMR	1501 Train:1126 Test:375	Train: 49.5 Test:50.6	Train: 54.4 Test: 54.7	SBP < 90	within 15 min after induction of spinal anesthesia	ANN simplified ANN LR	14	NP	ANN
Lu, 2023 [55]	China	Not specified	Local EMR and Registry	393,679 EMR: 387,291 Registry: 6388	NP	NP	NP	NP	composite multi-attention	7	Training/ validation/ testing	NA
Lee, 2019 [52]	USA	Both	Local EMR	224	NP	NP	(1) MAP decrease of > 40% from preinduction and postinduction MAP < 70 mmHg or (2) MAP < 60 mmHg	Post-induction	DNN LR	24	leave-one-out cross validation (LOO)	waveform only DNN
Shi, 2023 [57]	China	Non-cardiac	Registry	1378	NP	NP	MAP < 65	NP	NN (ResNet-BiLSTM + Multitask Learning + Attention Mechanism)	NP	Training/ validation/ testing with bootstrapping (over-sampling)	NA
Yoshimura, 2022 [59]	Japan	Non-cardiac surgery	Local EMR	1956	NP	43%	MAP < 55	Post-induction	DNN	49	K-fold validation (K = 10)	DNN
Feld, 2023 [53]	USA	Non-cardiac surgery (Neurosurgical procedures)	Local EMR	1005	median age = 57	71%	MAP < 65	Throughout surgery	LR, XGBoost	5, 15	K-fold validation (K = 5)	XGBoost
Hwang, 2023 [58]	South Korea	Not specified	Registry	Internal: 3278 External: 10,454	Internal: 59.4 External: 58.2	VitalDB: 55.3% AMC: 54%	MAP < 65	NP	NN	NP	Training/ internal validation/ external validation	NN
Kim, 2023 [60]	USA	Not specified	Registry	7062 train: 5021 test: 2041	NP	NP	MAP < 65	Throughout surgery	DNN	NP	Training/testing	DNN

*Abbreviations*:

*NP: not provided/ NA: not applicable*

*NN: neural networks DNN: deep neural network*

*ANN: artificial neural network*

*CNN: convolutional neural network*

*RNN: recurrent neural network*

*LR: logistic regression*

*RF: random forest*

*XGBoost: extreme gradient boosting*

*GBM: gradient boosting machine*

*LGBM: light gradient boosting machine*

*SVM: support vector machine*

*LDA: linear discriminant analysis*

*KNN: K-nearest neighbor*

*NB: Naïve Bayes*

*GP: Gaussian process*

*BGLM: Bayesian generalized linear models*

*BNN: Bayesian neural network*

*BNNMR: Bayesian neural network with multivariate mixed response*

*ABP: arterial blood pressure*

*ECG: electrocardiogram*

*EEG: electroencephalogram*

#### AUROC

**Fig. 6:**
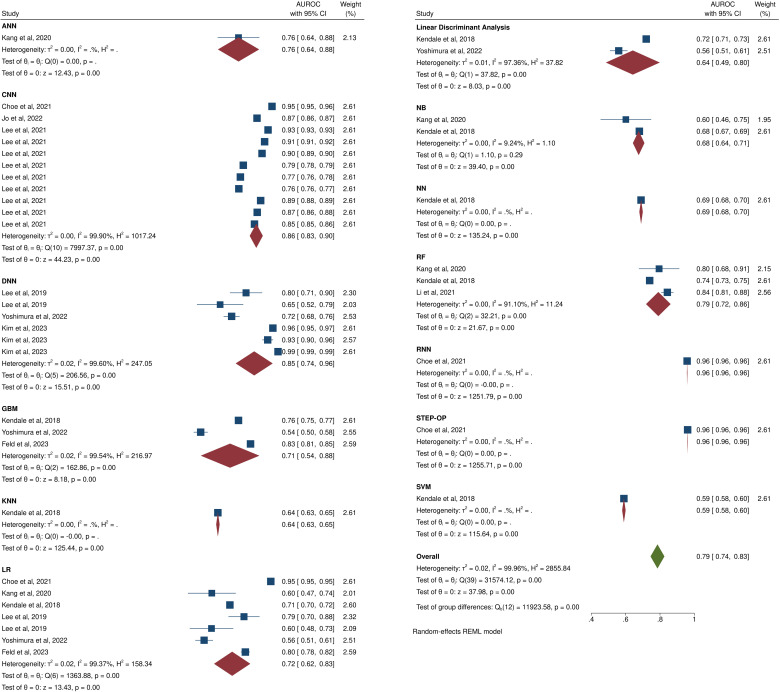
Meta-analysis of the AUROC of non-hypotension prediction index artificial intelligence models for the prediction of intraoperative hypotension stratified by the type of algorithm utilized. The overall AUROC of all the models for predicting intraoperative hypotension is presented at the bottom of the figure

10 of the 21 non-HPI studies [9, 41, 44, 45, 48, 49, 52–54, 60] had reported the AUROC of their AI models and were subgroup meta-analyzed by the algorithm employed in their models. Our meta-analysis found an AUROC of 0.79 (95CI: 0.74, 0.83) when all algorithms were pooled together, with substantial heterogeneity (I^2^ = 99%; Fig. 6). Sensitivity analysis showed our findings to be stable (data not shown) yet significant publication bias was present (Egger’s test *p*-value < 0.01; Additional file 1: Figure S6). In addition, subgroup analyses based on the definition of hypotension revealed that significantly higher AUROC values are achieved when IOH is defined as MAP < 65, while other definitions did not differe significantly in comparison to one another. Additionally high heterogeneity was present in each subgroup (Additional file 2: Figure S7).

### Quality assessment

**Fig. 7:**
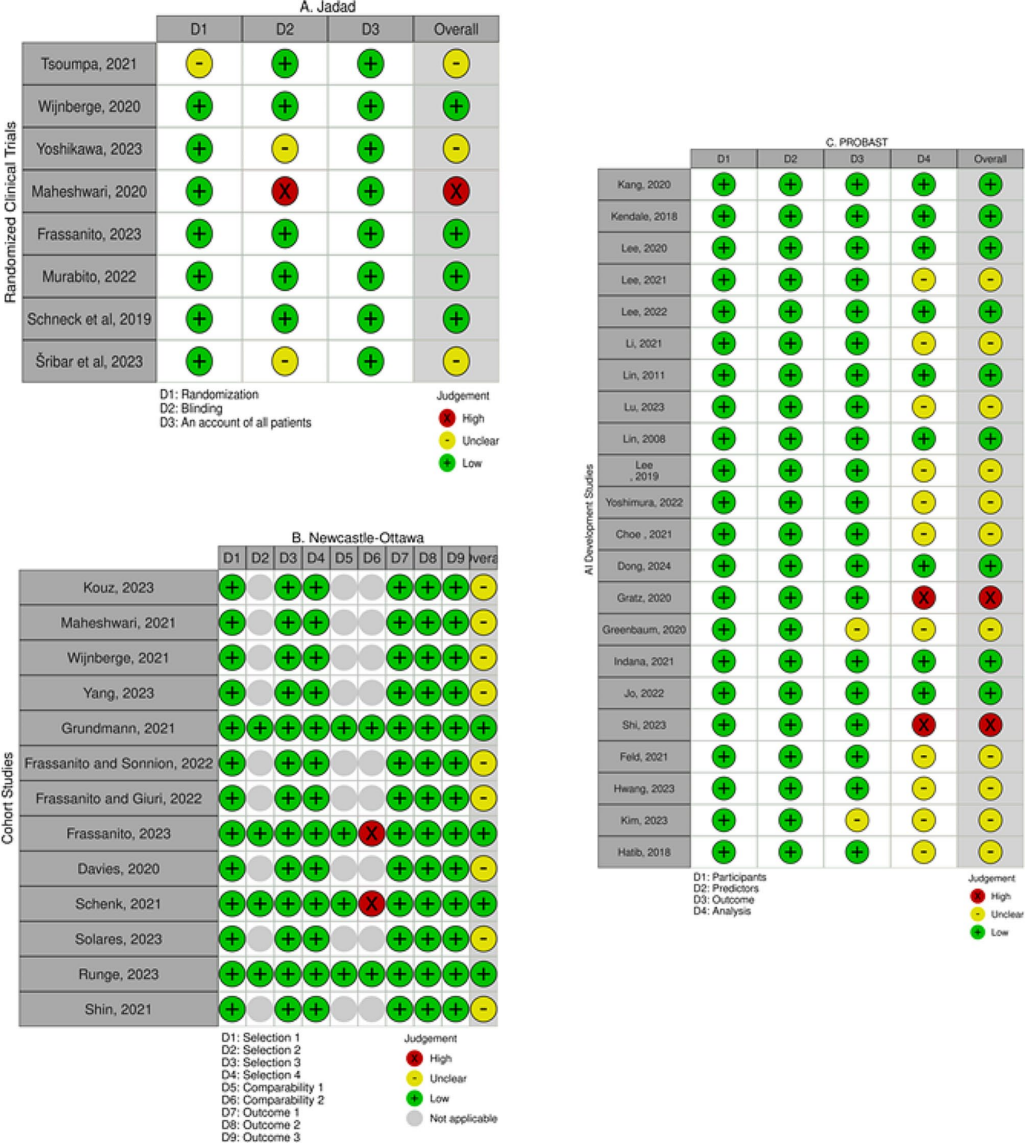
The quality of the included studies accoding to (**A**) the Jadad scale (RCTs), (**B**) the Newcastle-Ottawa scale (Cohort studies), and (**C**) the PROBAST tool (AI development studies)

Among the HPI studies, there were 13 cohort studies, 4 of which were judged to be of high quality, and the remaining 9 were assessed to be of unclear quality due to the absence of control groups. Out of the 8 randomized controlled trials, 4 had excellent quality according to the Jadad scale while there were some concerns about 3 studies and one had low quality. The HPI development study was also of unclear quality due to lack of information about the analyses conducted to develop the model (Fig. 7). Subgroup analyses of the HPI studies revealed that none of the measured outcomes _namely 5-, 10-, and 15-minute AUROCs, TWA-MAP < 65, duration of intraoperative hypotension, and AUT-MAP < 65_ were significantly different among studies with different qualities (Additional file 2: Figures S8-S13).

Among the non-HPI studies, 9 were judged to be high quality, 10 were of unclear quality and 2 had low quality. The main cause of unclear quality among the studies was the unclear status they received in the analysis domain of the PROBAST tool (Fig. 7). Subgroup analyses revealed that high-quality studies obtained significantly lower AUROCs compared to those of unclear quality (Additional file 2: Figure S14).

## Discussion

Intraoperative hypotension is a common phenomenon and has been associated with major postoperative complications such as major adverse renal [62], neurological [63], and cardiac [64] events and a high rate of mortality [65]. Despite these remarkable negative sequelae, the current management of IOH is predominantly reactive and often results in losing valuable time and higher IOH periods [6, 35, 49]. To remedy this issue, proactive approaches such as prediction models_ specifically those employing AI_ have recently been developed and tested in the relevant literature. Our study is the first systematic review and meta-analysis of the performance of AI models for the prediction of IOH. We found that one specific AI model, HPI, has been studied in 21 RCTs and cohort studies that externally validated it, and hence decided to evaluate it separately. In total, HPI was shown to significantly outperform the in-house protocol for predicting hypotension by reducing the cumulative duration of IOH per patient, TWA-MAP < 65, and AUT-MAP < 65. Furthermore, the pooled AUROC of HPI among the studies was 0.89, with the model demonstrating a statistically similar performance 5, 10, and 15 min before each hypotension event. In addition, 22 studies reported on the development of AI models other than HPI. Collectively, the non-HPI models achieved an AUROC of 0.79, with models incorporating recurrent, convolutional, and deep neural networks performing the best, and SVM models performing the worst.

### Hypotension prediction index

Regarding HPI, the results of our analyses are in accordance with a previous systematic review of RCTs evaluating the performance of HPI during non-cardiac surgeries. Li et al. included 5 studies, representing 461 patients, and ultimately found that the median differences of medians indicated an improvement in hypotension-related endpoints such as duration, incidence, percentage, TWA-MAP < 65, and AUT-MAP < 65 [66]. In contrast, our study was not limited to RCTs in order to gather the limited evidence in this novel subject matter more effectively, and as a result, we were also able to meta-analyze the AUROCs obtained by external validation studies of HPI, demonstrating its excellent predictive ability, particularly compared to other LR-based models. In addition, while HPI was originally designed based on invasive arterial line waveforms, many of the included reports have utilized it in combination with non-invasive arterial pressure waveforms and achieved comparable results [22, 23, 27, 28], expanding its applicability to potentially any patient undergoing surgery. It is also noteworthy that while the original HPI model was developed to predict IOH in noncardiac surgeries, Shin et al. conducted a cohort study testing the feasibility of its use in patients undergoing cardiac surgery requiring cardiopulmonary bypass, and concluded that HPI predicted IOH with a high degree of sensitivity and specificity [32]. As these results are promising, we recommend further research be conducted to examine HPI’s efficacy in cardiac surgeries.

### Non-HPI AI models

With respect to the non-HPI studies, we observed an excellent prediction ability among the deep learning algorithms, such as RNN, DNN, and CNN, with the highest AUROC achieved by STEP-OP [49], a model incorporating CNN and RNN (AUROC = 0.96). In general, deep learning has shown the potential to achieve more precise predictions than traditional ML in many areas of medical research [67], as it employs layers of neurons_as opposed to manual feature extraction in traditional ML_ and thus detects more abstract and generalized connections in the data [68]. As a result, it has been speculated that deep-learning algorithms may be able to detect subtle heralding changes in the arterial waveform which could be overlooked when represented as features in traditional ML models such as HPI [49]. Overall, although our meta-analysis demonstrated that HPI is very effective and indeed a formidable opponent for other prediction models, the model has been developed using LR [12], and non-HPI deep learning models hold the promise of even more accurate projections in the future.

In addition, our subgroup analyses revealed that studies employing a cut-off of MAP < 65 mm Hg for the definition of IOH performed significantly better in comparison to reports using other definitions. This finding is consistent with previous findings, as HPI also uses this definition of IOH [12] and has achieved excellent results by doing so, as evident by our analyses. Consequently, it seems that MAP < 65 mm Hg can be deemed as the best definition for IOH for the design of future IOH prediction models.

### Limitations

Our study has several limitations. While the analyses of the HPI studies were not heterogeneous, a high degree of heterogeneity was observed among the analyses of non-HPI models, and could not be explained with sub-group analyses based on AI model types or the definitions of IOH. We believe this heterogeneity can be due to the diverse nature of surgeries included, population makeup variations, and _in the cases of traditional ML models_differences in feature extraction. Second, while we were able to perform qualitative synthesis on 10 of the non-HPI studies, 12 of them could not be included due to either not reporting their AUROC and/or its confidence interval. In addition, many of the non-HPI studies were not of high quality, mainly due to underreporting in the analyses they performed to develop the model. We suggest that moving forward, all AI development studies meticulously mention the steps they take to develop the model, and also report AUROC in order to be eligible for inclusion in quantitative syntheses of meta-analysis studies. Further, to aid the design of future studies, it is advisable that features with high predictive values be discussed by the authors more extensively. Finally, while the results of our analyses regarding HPI are promising, the number of patients included in the studies is low and the study designs are far from robust. Additionally, Li et al. [66] found, the grade of evidence regarding the associated outcomes is poor. We recommend more RCTs with larger sample sizes and higher quality be conducted to remedy this issue.

## Conclusion

Artificial intelligence prediction models of intraoperative hypotension hold the potential to fundamentally change the way we treat this perilous condition. HPI, the first commercially available AI prediction model of IOH, demonstrated an excellent ability to predict hypotensive episodes and hence reduce the duration of hypotension. Other AI models, particularly those based on deep learning methods also demonstrated a great ability to predict IOH, while their capacity to reduce IOH-related indices such as duration remains unclear. This systematic review provides a comprehensive overlook of these models, serving as a stepping stone for future studies that may be conducted in this field.

### Electronic supplementary material

Below is the link to the electronic supplementary material.

**Additional file 1: Table S1:** Inclusion and exclusion criteria of HPI studies. **Table S2:** Characteristics of the non-HPI studies.

Additional file 2: **Figure S1:** Sensitivity analysis for the meta-analysis of the duration of intraoperative hypotension between hypotension prediction index-guided participants and participants receiving standard in-house protocols. **Figure S2**: Funnel plot for assessing the publication bias of our meta-analysis of the duration of intraoperative hypotension between hypotension prediction index-guided participants and participants receiving standard in-house protocols. The plot is asymmetric. **Figure S3**: Sensitivity analysis for the meta-analysis of the time-weighted average of hypotension (MAP < 65 mmHg) between hypotension prediction index-guided participants and participants receiving standard in-house protocols. **Figure S4**: Funnel plot for assessing the publication bias of our meta-analysis of the time-weighted average of hypotension (MAP < 65 mmHg) between hypotension prediction index-guided participants and participants receiving standard in-house protocols. The plot is asymmetric. **Figure S5**: Sensitivity analysis for the meta-analysis of the area under the threshold for hypotension (MAP < 65 mmHg) between hypotension prediction index-guided participants and participants receiving standard in-house protocols. **Figure S6**: Funnel plot for assessing the publication bias of our meta-analysis of the area under the receiver operating curve for non-hypotension prediction index artificial intelligence models for the prediction of intraoperative hypotension. The plot is asymmetric. **Figure S7**: Sub-group analysis of the AUROC meta-analysis for non-HPI studies based on the cut-off used to define hypotension. **Figure S8**: Sub-group analysis of the AUROC meta-analysis for HPI studies 5 minutes prior to intraoperative hypotension based on study quality and risk of bias. **Figure S9**: Sub-group analysis of the AUROC meta-analysis for HPI studies 10 minutes prior to intraoperative hypotension based on study quality and risk of bias. **Figure S10**: Sub-group analysis of the AUROC meta-analysis for HPI studies 15 minutes prior to intraoperative hypotension based on study quality and risk of bias. **Figure S11**: Sub-group analysis of the AUT-MAP < 65 meta-analysis for HPI studies based on study quality and risk of bias. **Figure S12**: Sub-group analysis of the duration of intraoperative hypotension meta-analysis for HPI studies based on study quality and risk of bias. **Figure S13**: Sub-group analysis of the TWA-MAP < 65 meta-analysis for HPI studies based on study quality and risk of bias. **Figure S14**: Sub-group analysis of the AUROC meta-analysis for non-HPI studies based on study quality and risk of bias.

Supplementary Material 3

Supplementary Material 4

## Data Availability

All the supporting data are included in the study.

## Abbreviations

IOH: Intraoperative hypotension
AI: Artificial intelligence
HPI: Hypotension prediction index
TWA-MAP < 65: Time weighted average of mean arterial pressure < 65
AUT-MAP: Area under the threshold of mean arterial pressure
AUROC: Area under the receiver operating characteristics curve
CPB: Cardiopulmonary bypass
PRISMA: Preferred Reporting Items for Systematic Reviews and Meta-Analyses
SMD: Standardized mean difference
CI: Confidence interval
NP: Not provided
NA: Not applicable
NN: Neural networks
DNN: Deep neural network
ANN: Artificial neural network
CNN: Convolutional neural network
RNN: Recurrent neural network
LR: Logistic regression
RF: Random forest
XGBoost: Extreme gradient boosting
GBM: Gradient boosting machine
LGBM: Light gradient boosting machine
SVM: Support vector machine
LDA: Linear discriminant analysis
KNN: K-nearest neighbor
NB: Naïve Bayes
GP: Gaussian process
BGLM: Bayesian generalized linear models
BNN: Bayesian neural network
BNNMR: Bayesian neural network with multivariate mixed response
ABP: Arterial blood pressure
ECG: Electrocardiogram
EEG: Electroencephalogram
EMR: Electronic medical records
