# Supplementary material for: Predictive ability of hypotension prediction index and machine learning methods in intraoperative hypotension: a systematic review and meta-analysis
Source: J Transl Med. 2024 Aug 5;22:725. doi: 10.1186/s12967-024-05481-4 (PMC11302102; doi:10.1186/s12967-024-05481-4)
Supplement: Supplementary file 1 — Additional file 1: Table S1: Inclusion and exclusion criteria of HPI studies. Table S2: Characteristics of the non-HPI studies. [file 12967_2024_5481_MOESM1_ESM.docx]

**Additional file 1**

**Predictive Ability of Hypotension Prediction Index and Machine Learning Methods in Intraoperative Hypotension: a Systematic Review and Meta-analysis**

| **Supplementary Tables** |  |
| --- | --- |
| Table S1 | *page 2* |
| Table S2 | *page 5* |
| References | *page 7* |

Table S1 Inclusion and exclusion criteria of HPI studies

| Author and year | Inclusion critera | Exclusion criteria |
| --- | --- | --- |
| Frassanito, 2023  (1) | Patients≥18 years old with American Society of Anesthesiologists (ASA) physical status II-IV, scheduled to major gynecologic oncologic surgery with expected duration>2 h under general anesthesia and planned continuous invasive blood pressure monitoring. | Significant cardiac arrhythmias, such as permanent atrial fibrillation, aortic regurgitation, coagulation disorders, emergency surgery, preoperative infection, the requirement of dialysis, contraindication to radial artery cannulation and patient’s refusal of the treatment of personal data |
| Frassanito, 2022  (2) | All patients scheduled for GOS who had intact complete data sets available for analysis were enrolled in the study. | Significant cardiac arrhythmias or aortic regurgitation, permanent atrial fibrillation, coagulation disorders, emergency surgery, preoperative infection and patient’s refusal to treatment of personal data |
| Frassanito, 2023  (3) | NP | NP |
| Frassanito, 2022  (4) | All patients who had complete data sets available for analysis were enrolled in the study. | <18 years of age, an American Society of Anesthesiologists (ASA) physical status ≥III, significant cardiac arrhythmias or aortic regurgitation, pregnancy-induced hypertension, preeclampsia, obesity (BMI >35 kg/m^2^), any fetal complication, contraindications to neuraxial block, emergency surgery, preoperative infection, patient’s refusal. |
| Davies, 2020  (5) | Subjects undergoing major surgery (major abdominal, vascular, or off-pump coronary artery bypass surgery) requiring arterial cannulation for blood pressure and/or cardiac output (CO) measuring and who had intact complete data sets available for analysis | NP |
| Grundmann, 2021  (6) | Elective major abdominal surgery (e.g., cystectomy, pancreaticoduodenectomy, and cytoreductive surgery/HIPEC), age > 18 years, anticipated duration of surgery > 120 minutes, and need of invasive blood pressure monitoring using an arterial line. | Exclusion criteria were patients not in sinus rhythm, ejection fraction < 30%, severe aortic valve stenosis, emergency surgery, acute myocardial ischemia, anticipated duration of surgery < 120 minutes and contraindication for an arterial line |
| Murabito, 2022  (7) | Adult patients (≥18 years old) undergoing elective laparotomic major general surgery under general anesthesia if an intraoperative continuous invasive blood pressure monitoring was planned with a MAP target of 65. | Patients for whom the attending anesthesiologists requested a different MAP target were excluded. Other exclusion criteria were: emergency surgery, patients with cardiac failure or cardiac shunts, |
| Kouz, 2023  (8) | Consenting adults (>18 yr) who were scheduled for elective major noncardiac surgery under general anesthesia that was expected to last at least 120 min and in whom intra-arterial pressure and HPI-software monitoring were planned for clinical management. | Patients having emergency surgery, nephrectomy, and liver or kidney transplantation; patients with atrial fibrillation or sepsis patients with ASA physical status classification 5 or 6; patients who were not able to understand the nature, significance, and scope of the investigation; pregnant women; patients without signed informed consent; and patients participating in interventional trials |
| Maheshwari, 2021  (9) | Adults aged 45 years or older designated ASA physical status 3 or 4 who had moderate-to-high-risk non-cardiac surgery with general anesthesia | Patients were excluded if the attending anesthesiologist determined that invasive arterial monitoring was needed. Patients were also excluded when there was more than a 10% discrepancy in preoperative MAP between the arms, or if the expected duration of surgery exceeded 2 h. |
| Maheshwari, 2020  (10) | Adults 45 yr old or greater who were designated American Society of Anesthesiologists (ASA) physical status III or IV and had moderate- or high-risk noncardiac surgery as defined by the responsible anesthesiologist and planned invasive blood pressure monitoring. All had general anesthesia expected to last more than 2 h and planned overnight hospitalization. | Urgent/ emergency procedures, patients with known clinically important intracardiac shunts, moderate to severe valvular disease, need of tidal volume less than 8ml/kg of ideal body weight during surgery, current persistent atrial fibrillation, congestive heart failure with ejection fraction less than 35%, and neurosurgical procedures. |
| Runge, 2023  (11) | All datasets that presented complete preoperative, intraoperative and postoperative data from patients undergoing moderate- or high-risk non-cardiac surgery in the departments of urology, general surgery, vascular surgery, and gynecology | NP |
| Schenk, 2021  (12) | Subjects 18 yr old scheduled for elective noncardiac surgery under general anesthesia with a target MAP 65 mm Hg, using continuous invasive BP monitoring. | NP |
| Schneck, 2019  (13) | an age ≥ 18 years and the need for elective total hip arthroplasty (THA) under general anesthesia. | Patients undergoing THA under regional anesthesia were excluded in order to allow the use of stroke volume variation as preload indicator and to achieve comparable hemodynamic reactions. Exclusion criteria included ASA>III, pregnancy or nursing, participation in another interventional study, contraindications for invasive blood pressure monitoring, severe chronic kidney disease (need for dialysis), and/or coagulopathy. |
| Shin, 2021  (14) | All patients older than 18 years and scheduled for elective cardiac surgery requiring CPB | NP |
| Šribar, 2023  (15) | Participants are patients over 18 years of age which were scheduled for elective major thoracic procedure (lung resection, pleurectomy or resection of the esophagus) with planned thoracotomy and intraoperative period of one lung ventilation with planned postoperative admission to the ICU. | Exclusion criteria were persistent atrial fibrillation, structural heart defects (shunting or moderate to severe valvular anomalies), preoperative serum hemoglobin levels<120 g/L and severe heart failure classified as New York Heart Association (NYHA) grade IV. |
| Solares, 2023  (16) | Adult patients undergoing major elective or urgent trauma/orthopedic, neurosurgical, vascular, or intraabdominal surgery with a moderate-to-high risk of bleeding. | Exclusion criteria for the analysis were restricted to the following cases: Patients<18 years or with a surgical duration of less than 1-h, patients undergoing cardiac surgery, solid organ transplant, organ donor subjects, or patients requiring dialysis and/or with a glomerular filtration rate<15 ml/min/1.73 m2 |
| Tsoumpa, 2021  (17) | Patients aged 18 or older undergoing elective non-cardiac surgery that required a minimum duration of 2 h under general anesthesia, with need for continuous invasive blood pressure monitoring intraoperatively. | The exclusion criteria included a MAP target other than 65 mmHg according to the attending anesthesiologist. Patients with significant hypotension as measured before surgery, with known left or right cardiac failure, known arrythmias (e.g., atrial fibrillation), cardiac shunts, severe aortic stenosis or the need for dialysis were also excluded. If surgery included clamping of the aorta or Pringle’s maneuver, the patient was not eligible for the trial. Finally, emergency procedures were also excluded from the trial. |
| Wijnberge, 2020  (18) | Adult patients (≥18 years old) scheduled to undergo an elective noncardiac surgical procedure under general anesthesia with need for continuous invasive blood pressure monitoring per arterial line were included. | Patients for whom the attending anesthesiologists requested a target MAP higher or lower than 65 mm Hg ; patients undergoing emergency surgery; patients with cardiac failure, severe cardiac shunts, severe aortic stenosis, or severe cardiac arrhythmias were excluded in accordance with the summary of product characteristics of the early warning system; patients with hypotension (MAP <65 mm Hg) before surgery and patients requiring dialysis ; patients planned to undergo liver surgery or vascular surgery |
| Wijnberge, 2021  (19) | Patients scheduled for noncardiac surgery and procedures with a planned duration of more than 2 h, allowing time for collection of a sufficient amount of high-quality data | Participants were only excluded if there were technical problems or when strong local vasoconstriction, such as cold fingers, prevented cNIBP measurement. |
| Hatib, 2018  (20) | age> 18 | NP |
| Yang, 2023  (21) | Patients older than 20 years who were scheduled for living donor liver transplantation | Patients with arrhythmias and those with a pacemaker |
| Yoshikawa, 2023  (22) | Adult patients (≥ 45 years old) scheduled for non-cardiac surgery with arterial pressure monitoring. | Patients with severe heart valve disease, arrhythmia, intracardiac shunt, chronic hemodialysis due to end-stage kidney disease, emergency surgery, vascular surgery, hepatic surgery, neurosurgery, thoracic surgery, and prone positioning during surgery. |

Table S2 Characteristics of the non-HPI studies

| First author and year | Patient selection | | Model details | | |
| --- | --- | --- | --- | --- | --- |
|  | Inclusion criteria | Exclusion criteria | Feature selection | 5 most predictive variables | Missing data strategy |
| Choe, 2021 (23) | NP | NP | NA | NP | Data cleaning |
| Dong, 2024 (24) | Age>18  undergoing hysterectomy | NP | NP | NP (supplement unavailable) | Imputation (principal component analysis) |
| Gratz, 2020 (25) | Patients (> 34 weeks gestation) with an American Society of Anesthesiologists status II who were undergoing elective C/S under spinal anesthesia | 1. No spinal injection 2. Short pre-injection data session 3. data sessions compromised due to motion artifacts | Predetermined by authors | Arterial stiffness | NA |
| Greenbaum, 2020 (26) | patients 18 to 65 years of age | Laparoscopic procedures were excluded | Predetermined by authors | NP | NP |
| Inada, 2021 (27) | Age>18 | NP | Recursive feature elimination | 1. Age 2. Anesthesia method (combined general anesthesia and epidural anesthesia, or general anesthesia and spinal anesthesia) 3. Preoperative systolic blood pressure | Interpolation/ exclusion |
| Jo, 2022 (28) | (1) adults (age>18); (2) administered general anesthesia; and (3) undergone non-cardiac surgery | (1) any missing monitoring for ABP, ECG, and EEG waveforms; and (2) cases containing false events or non-events due to poor signal quality shown as | ResNet | NP | Data cleaning |
| Kang, 2020 (29) | Adult patients (age > = 18 years) who underwent laparoscopic cholecystectomy | NP | Recursive feature elimination | 1. Lowest SBP 2. Lowest MAP 3. Mean SBP before tracheal intubation | NP |
| Kendale, 2018 (30) | Age>12 | NP | Recursive feature elimination | 1. First mean arterial pressure  2. Age 3. Body Mass Index | NP |
| Lee, 2020 (31) | Adult patients (age ≥18 years) who underwent laparoscopic cholecystectomy under general anesthesia | NP | Statistics (t-test and Wilcoxon rank-sum for continuous variables; chi-squared and Fisher's exact test for categorical variables) | 1. Minimum non-invasive SBP 2. Mean non-invasive SBP 3. Mean non-invasive MAP | NP |
| Lee, 2021 (32) | Only patients with sufficient information | NP | NP | NP | NP |
| Lee, 2022 (33) | Adult patients (age > = 18 years) who underwent laparoscopic cholecystectomy | NP | Domain knowledge (statistics, peak analysis, Fourier transform and wavelet transform were used to determine the best features in each category) | 1. Mean BP 2. Root sum square of BP 3. Root mean square of BP | NP |
| Li, 2021 (34) | NP | Those who were younger than 18 years of age, had abandoned surgical treatment, and those with incomplete preoperative data | Embedded (RF) | 1. Mean PB 2. Age 3. Body Mass index | NP |
| Lin, 2011 (35) | NP | Patients who underwent inhalation  induction of anesthesia, patients who had received more than 1 of the usual drugs to induce anesthesia (i.e., thiopental, propofol, or etomidate), or rarely used regimens (e.g., ketamine) | Predetermined by authors | 1. Age 2. Gender 3. Emergency status | NP |
| Lin, 2008 (36) | NP | patients with missing data | Mann–Whitney U-test, chi-square or Fisher's exact test + LR | NP | NP |
| Lu, 2023 (37) | NP | NP | multi-attention mechanism | NP | records with a missing rate  above 20% are remove from dataset |
| Lee, 2019 (38) | NP | Patients  with no induction time, no MAP 10 minutes after induction, negative time difference between surgical  start and induction, < 18 years of age, or no ABP waveform prior to induction | Predetermined by authors | NP | NP |
| Shi, 2023 (39) | NP | NP | ResNet | NP | NA |
| Yoshimura, 2022 (40) | Noncardiac and non-obstetric surgery patients (aged >18 years) | Patients who had not undergone preoperative echocardiography within one preoperative month, or who underwent echocardiography other than for preoperative purposes | SHAP method | 1. Ascending aorta diameter 2. Tricuspid regurgitation pressure gradient 3. Heart rate | Substituted with normal data |
| Feld, 2023 (41) | NP | NP | NP | 1. Current value of MAP 2. 5-minute exponential moving averages of MAP 3. 15-minute exponential moving averages of MAP | linear interpolation and Imputation |
| Hwang, 2023 (42) | NP | NP | NP | NP | NP |
| Kim, 2023 (43) | NP | NP | NP | NP | NP |

Abbreviations:
BP: blood pressure
SBP: systolic blood pressure
MAP: mean arterial pressure

**References:**

1. Frassanito L, Giuri PP, Vassalli F, Piersanti A, Garcia MIM, Sonnino C, et al. Hypotension Prediction Index guided Goal Directed therapy and the amount of Hypotension during Major Gynaecologic Oncologic Surgery: a Randomized Controlled clinical Trial. J Clin Monit Comput. 2023;37(4):1081-93.

2. Frassanito L, Giuri PP, Vassalli F, Piersanti A, Longo A, Zanfini BA, et al. Hypotension Prediction Index with non-invasive continuous arterial pressure waveforms (ClearSight): clinical performance in Gynaecologic Oncologic Surgery. J Clin Monit Comput. 2022;36(5):1325-32.

3. Frassanito L, Giuri PP, Vassalli F, Piersanti A, Zanfini BA, Catarci S, et al. Noninvasive hypotension Prediction Index versus continuous blood pressure monitoring and intraoperative hypotension. Minerva Anestesiol. 2023;89(6):603-5.

4. Frassanito L, Sonnino C, Piersanti A, Zanfini BA, Catarci S, Giuri PP, et al. Performance of the Hypotension Prediction Index With Noninvasive Arterial Pressure Waveforms in Awake Cesarean Delivery Patients Under Spinal Anesthesia. Anesth Analg. 2022;134(3):633-43.

5. Davies SJ, Vistisen ST, Jian Z, Hatib F, Scheeren TWL. Ability of an Arterial Waveform Analysis-Derived Hypotension Prediction Index to Predict Future Hypotensive Events in Surgical Patients. Anesth Analg. 2020;130(2):352-9.

6. Grundmann CD, Wischermann JM, Fassbender P, Bischoff P, Frey UH. Hemodynamic monitoring with Hypotension Prediction Index versus arterial waveform analysis alone and incidence of perioperative hypotension. Acta Anaesthesiol Scand. 2021;65(10):1404-12.

7. Murabito P, Astuto M, Sanfilippo F, La Via L, Vasile F, Basile F, et al. Proactive Management of Intraoperative Hypotension Reduces Biomarkers of Organ Injury and Oxidative Stress during Elective Non-Cardiac Surgery: A Pilot Randomized Controlled Trial. J Clin Med. 2022;11(2).

8. Kouz K, Monge García MI, Cerutti E, Lisanti I, Draisci G, Frassanito L, et al. Intraoperative hypotension when using hypotension prediction index software during major noncardiac surgery: a European multicentre prospective observational registry (EU HYPROTECT). BJA Open. 2023;6:100140.

9. Maheshwari K, Buddi S, Jian Z, Settels J, Shimada T, Cohen B, et al. Performance of the Hypotension Prediction Index with non-invasive arterial pressure waveforms in non-cardiac surgical patients. J Clin Monit Comput. 2021;35(1):71-8.

10. Maheshwari K, Shimada T, Yang D, Khanna S, Cywinski JB, Irefin SA, et al. Hypotension Prediction Index for Prevention of Hypotension during Moderate- to High-risk Noncardiac Surgery. Anesthesiology. 2020;133(6):1214-22.

11. Runge J, Graw J, Grundmann CD, Komanek T, Wischermann JM, Frey UH. Hypotension Prediction Index and Incidence of Perioperative Hypotension: A Single-Center Propensity-Score-Matched Analysis. J Clin Med. 2023;12(17).

12. Schenk J, Wijnberge M, Maaskant JM, Hollmann MW, Hol L, Immink RV, et al. Effect of Hypotension Prediction Index-guided intraoperative haemodynamic care on depth and duration of postoperative hypotension: a sub-study of the Hypotension Prediction trial. Br J Anaesth. 2021;127(5):681-8.

13. Schneck E, Schulte D, Habig L, Ruhrmann S, Edinger F, Markmann M, et al. Hypotension Prediction Index based protocolized haemodynamic management reduces the incidence and duration of intraoperative hypotension in primary total hip arthroplasty: a single centre feasibility randomised blinded prospective interventional trial. J Clin Monit Comput. 2020;34(6):1149-58.

14. Shin B, Maler SA, Reddy K, Fleming NW. Use of the Hypotension Prediction Index During Cardiac Surgery. J Cardiothorac Vasc Anesth. 2021;35(6):1769-75.

15. Šribar A, Jurinjak IS, Almahariq H, Bandić I, Matošević J, Pejić J, et al. Hypotension prediction index guided versus conventional goal directed therapy to reduce intraoperative hypotension during thoracic surgery: a randomized trial. BMC Anesthesiol. 2023;23(1).

16. Solares GJ, Garcia D, Garcia MIM, Crespo C, Rabago JL, Iglesias F, et al. Real-world outcomes of the hypotension prediction index in the management of intraoperative hypotension during non-cardiac surgery: a retrospective clinical study. J Clin Monit Comput. 2023;37(1):211-20.

17. Tsoumpa M, Kyttari A, Matiatou S, Tzoufi M, Griva P, Pikoulis E, et al. The use of the hypotension prediction index integrated in an algorithm of goal directed hemodynamic treatment during moderate and high-risk surgery. Journal of Clinical Medicine. 2021;10(24).

18. Wijnberge M, Geerts BF, Hol L, Lemmers N, Mulder MP, Berge P, et al. Effect of a Machine Learning-Derived Early Warning System for Intraoperative Hypotension vs Standard Care on Depth and Duration of Intraoperative Hypotension during Elective Noncardiac Surgery: The HYPE Randomized Clinical Trial. JAMA - Journal of the American Medical Association. 2020;323(11):1052-60.

19. Wijnberge M, Van Der Ster BJP, Geerts BF, De Beer F, Beurskens C, Emal D, et al. Clinical performance of a machine-learning algorithm to predict intra-operative hypotension with noninvasive arterial pressure waveforms: A cohort study. Eur J Anaesthesiol. 2021;38(6):609-15.

20. Hatib F, Jian Z, Buddi S, Lee C, Settels J, Sibert K, et al. Machine-learning Algorithm to Predict Hypotension Based on High-fidelity Arterial Pressure Waveform Analysis. Anesthesiology. 2018;129(4):663-74.

21. Yang SM, Cho HY, Lee HC, Kim HS. Performance of the Hypotension Prediction Index in living donor liver transplant recipients. Minerva Anestesiol. 2023;89(5):387-95.

22. Yoshikawa Y, Maeda M, Kunigo T, Sato T, Takahashi K, Ohno S, et al. Effect of using hypotension prediction index versus conventional goal-directed haemodynamic management to reduce intraoperative hypotension in non-cardiac surgery: A randomised controlled trial. J Clin Anesth. 2023;93:111348.

23. Choe S, Park E, Shin W, Koo B, Shin D, Jung C, et al. Short-Term Event Prediction in the Operating Room (STEP-OP) of Five-Minute Intraoperative Hypotension Using Hybrid Deep Learning: Retrospective Observational Study and Model Development. JMIR Medical Informatics. 2021;9(9):e31311.

24. Dong Z, Chen X, Ritter J, Bai L, Huang J. American society of anesthesiologists physical status classification significantly affects the performances of machine learning models in intraoperative hypotension inference. J Clin Anesth. 2024;92:111309.

25. Gratz I, Baruch M, Takla M, Seaman J, Allen I, McEniry B, et al. The application of a neural network to predict hypotension and vasopressor requirements non-invasively in obstetric patients having spinal anesthesia for elective cesarean section (C/S). BMC Anesthesiol. 2020;20(1):98.

26. Greenbaum NR, White ST. Machine Learning Predicts Intraoperative Hypotension from End-Tidal Carbon Dioxide Measurement. Anesth Analg. 2020;130.

27. Inada R, Doi C, Yamasaki Y, Shigeno H, Seki H, editors. Intraoperative Hypotension Prediction System by Considering Personal Lifestyle and Medical History. 2021 IEEE International Conference on Pervasive Computing and Communications Workshops and other Affiliated Events, PerCom Workshops 2021; 2021.

28. Jo YY, Jang JH, Kwon JM, Lee HC, Jung CW, Byun S, et al. Predicting intraoperative hypotension using deep learning with waveforms of arterial blood pressure, electroencephalogram, and electrocardiogram: Retrospective study. PLoS ONE. 2022;17(8 August):e0272055.

29. Kang AR, Lee J, Jung W, Lee M, Park SY, Woo J, et al. Development of a prediction model for hypotension after induction of anesthesia using machine learning. PLoS ONE. 2020;15(4):e0231172.

30. Kendale S, Kulkarni P, Rosenberg AD, Wang J. Supervised Machine-learning Predictive Analytics for Prediction of Postinduction Hypotension. Anesthesiology. 2018;129(4):675-88.

31. Lee J, Woo J, Kang AR, Jeong YS, Jung W, Lee M, et al. Comparative analysis on machine learning and deep learning to predict post-induction hypotension. Sensors (Basel). 2020;20(16):1-21.

32. Lee S, Lee HC, Chu YS, Song SW, Ahn GJ, Lee H, et al. Deep learning models for the prediction of intraoperative hypotension. Br J Anaesth. 2021;126(4):808-17.

33. Lee S, Lee M, Kim SH, Woo J. Intraoperative Hypotension Prediction Model Based on Systematic Feature Engineering and Machine Learning. Sensors (Basel). 2022;22(9).

34. Li XF, Huang YZ, Tang JY, Li RC, Wang XQ. Development of a random forest model for hypotension prediction after anesthesia induction for cardiac surgery. World J Clin Cases. 2021;9(29):8729-39.

35. Lin CS, Chang CC, Chiu JS, Lee YW, Lin JA, Mok MS, et al. Application of an artificial neural network to predict postinduction hypotension during general anesthesia. Med Decis Making. 2011;31(2):308-14.

36. Lin CS, Chiu JS, Hsieh MH, Mok MS, Li YC, Chiu HW. Predicting hypotensive episodes during spinal anesthesia with the application of artificial neural networks. Comput Methods Programs Biomed. 2008;92(2):193-7.

37. Lu F, Li W, Zhou Z, Song C, Sun Y, Zhang Y, et al., editors. A Composite Multi-Attention Framework for Intraoperative Hypotension Early Warning. Proceedings of the 37th AAAI Conference on Artificial Intelligence, AAAI 2023; 2023.

38. Lee C, Cannesson M, Baldi P. Prediction of Postinduction Hypotension with Deep Learning. Anesth Analg. 2019;128.

39. Shi M, Zheng Y, Wu Y, Ren Q. Multitask Attention-Based Neural Network for Intraoperative Hypotension Prediction. Bioengineering. 2023;10(9).

40. Yoshimura M, Shiramoto H, Koga M, Morimoto Y. Preoperative echocardiography predictive analytics for postinduction hypotension prediction. PLoS ONE. 2022;17(11 November).

41. Feld S, Hippe DS, Miljacic N, Polissar NL, Newman SF, Nair BG, et al. A Machine Learning Approach for Predicting Real Time Risk of Intraoperative Hypotension in Traumatic Brain Injury. Anesth Analg. 2021;132(5S_SUPPL):514-5.

42. Hwang E, Park YS, Kim JY, Park SH, Kim J, Kim SH. Intraoperative Hypotension Prediction Based on Features Automatically Generated Within an Interpretable Deep Learning Model. IEEE Trans Neural Netw Learn Syst. 2023;Pp.

43. Kim S, Kwon S, Bovik AC, Markey MK, Cannesson M. Machine learning predicting hypotension in operating room: application of deep neural networks on continuous physiologic waveforms for early prediction of intraoperative hypotension. Anesth Analg. 2023;136(4):90-1.
