# Supplementary material for: Predictive ability of hypotension prediction index and machine learning methods in intraoperative hypotension: a systematic review and meta-analysis
Source: J Transl Med. 2024 Aug 5;22:725. doi: 10.1186/s12967-024-05481-4 (PMC11302102; doi:10.1186/s12967-024-05481-4)
Supplement: Supplementary file 2 — Additional file 2: Figure S1: Sensitivity analysis for the meta-analysis of the duration of intraoperative hypotension between hypotension prediction index-guided participants and participants receiving standard in-house protocols. Figure S2: Funnel plot for assessing the publication bias of our meta-analysis of the duration of intraoperative hypotension between hypotension prediction index-guided participants and participants receiving standard in-house protocols. The plot is asymmetric. Figure S3: Sensitivity analysis for the meta-analysis of the time-weighted average of hypotension (MAP < 65 mmHg) between hypotension prediction index-guided participants and participants receiving standard in-house protocols. Figure S4: Funnel plot for assessing the publication bias of our meta-analysis of the time-weighted average of hypotension (MAP < 65 mmHg) between hypotension prediction index-guided participants and participants receiving standard in-house protocols. The plot is asymmetric. Figure S5: Sensitivity analysis for the meta-analysis of the area under the threshold for hypotension (MAP < 65 mmHg) between hypotension prediction index-guided participants and participants receiving standard in-house protocols. Figure S6: Funnel plot for assessing the publication bias of our meta-analysis of the area under the receiver operating curve for non-hypotension prediction index artificial intelligence models for the prediction of intraoperative hypotension. The plot is asymmetric. Figure S7: Sub-group analysis of the AUROC meta-analysis for non-HPI studies based on the cut-off used to define hypotension. Figure S8: Sub-group analysis of the AUROC meta-analysis for HPI studies 5 minutes prior to intraoperative hypotension based on study quality and risk of bias. Figure S9: Sub-group analysis of the AUROC meta-analysis for HPI studies 10 minutes prior to intraoperative hypotension based on study quality and risk of bias. Figure S10: Sub-group analysis of the AUROC meta-analysis [file 12967_2024_5481_MOESM2_ESM.docx]

**Additional file 2**

**Predictive Ability of Hypotension Prediction Index and Machine Learning Methods in Intraoperative Hypotension: a Systematic Review and Meta-analysis**

| **Supplementary Figures** |  |
| --- | --- |
| Figure S1 | *page 2* |
| Figure S2 | *Page 3* |
| Figure S3 | *Page 4* |
| Figure S4 | *Page 5* |
| Figure S5 | *Page 6* |
| Figure S6 | *Page 7* |
| Figure S7 | *Page 9* |
| Figure S8 | *Page 10* |
| Figure S9 | *Page 11* |
| Figure S10 | *Page 12* |
| Figure S11 | *Page 13* |
| Figure S12 | *Page 14* |
| Figure S13 | *Page 15* |
| Figure S14 | *Page 16* |
| **References** | *Page 17* |

Figure S1: Sensitivity analysis for the meta-analysis of the duration of intraoperative hypotension between hypotension prediction index-guided participants and participants receiving standard in-house protocols.


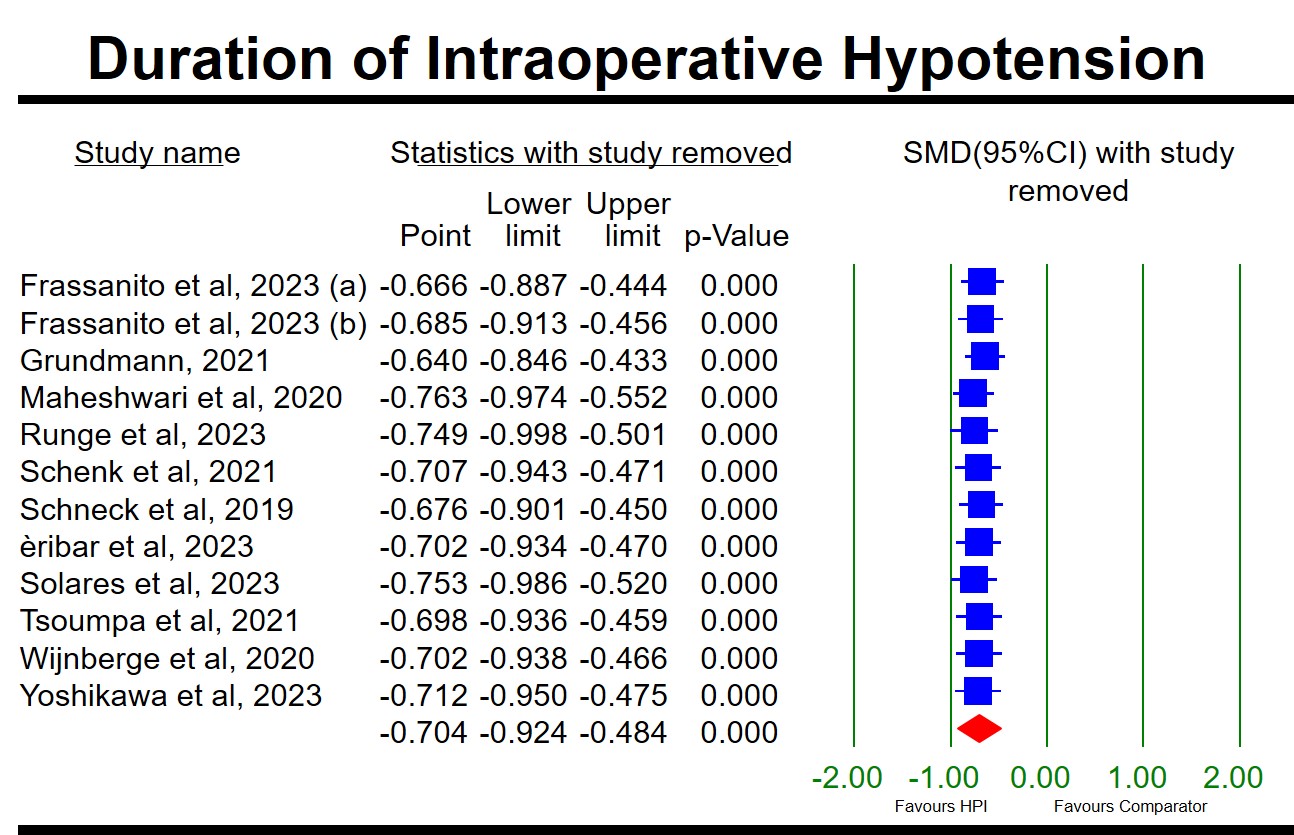


(a): (1)
(b): (2)

Figure S2: Funnel plot for assessing the publication bias of our meta-analysis of the duration of intraoperative hypotension between hypotension prediction index-guided participants and participants receiving standard in-house protocols. The plot is asymmetric.


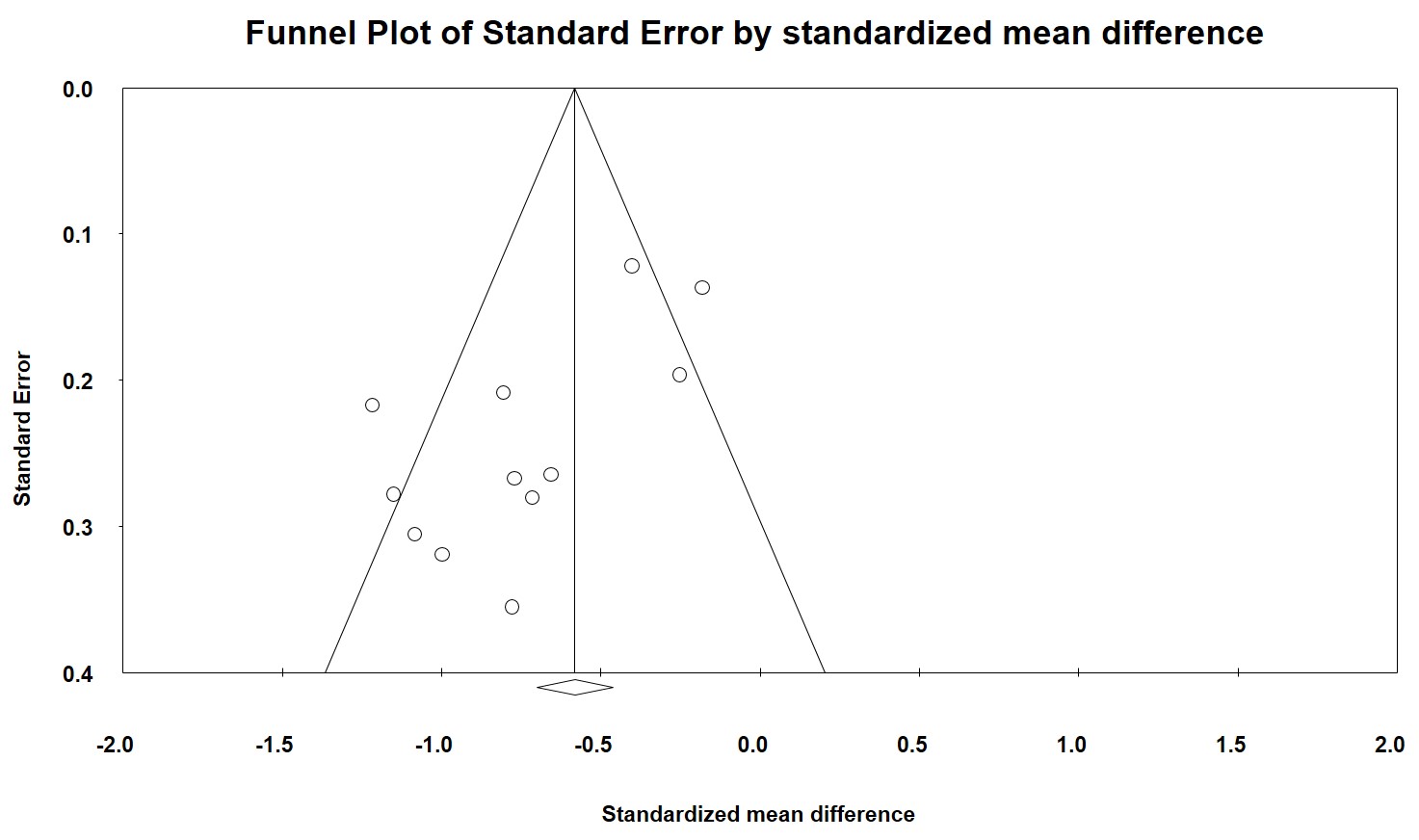


Figure S3: Sensitivity analysis for the meta-analysis of the time-weighted average of hypotension (MAP<65 mmHg) between hypotension prediction index-guided participants and participants receiving standard in-house protocols.


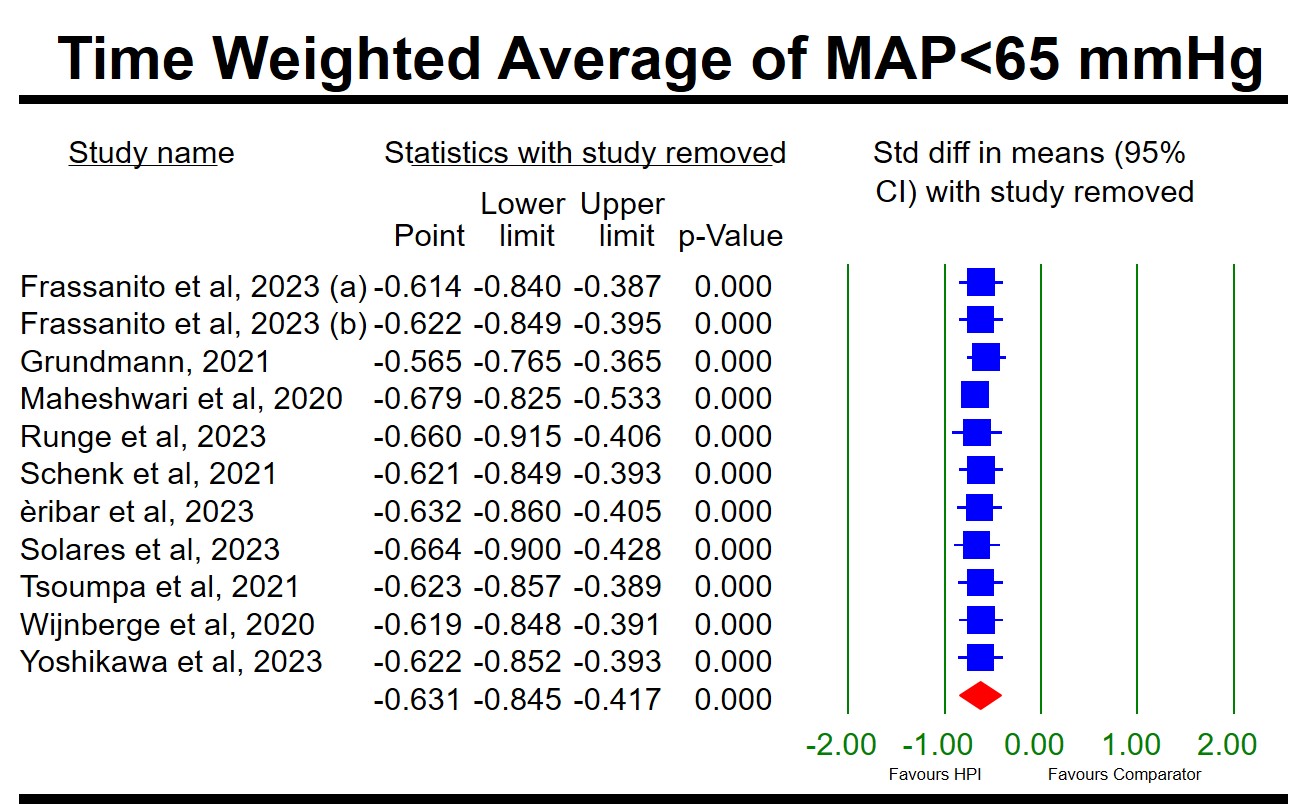


(a): (1)
(b): (2)

Figure S4: Funnel plot for assessing the publication bias of our meta-analysis of the time-weighted average of hypotension (MAP<65 mmHg) between hypotension prediction index-guided participants and participants receiving standard in-house protocols. The plot is asymmetric. **
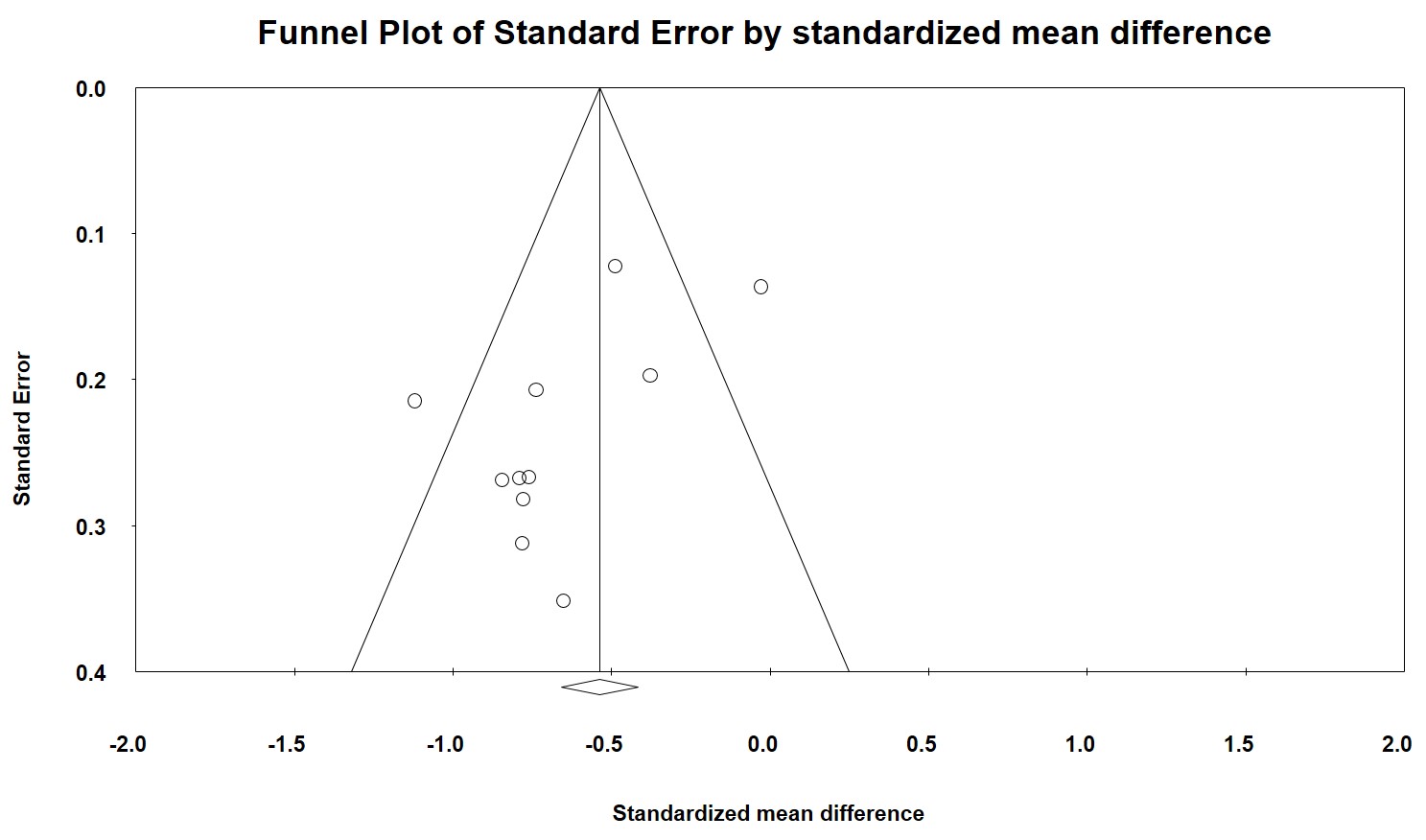
**

Figure S5: Sensitivity analysis for the meta-analysis of the area under the threshold for hypotension (MAP<65 mmHg) between hypotension prediction index-guided participants and participants receiving standard in-house protocols.


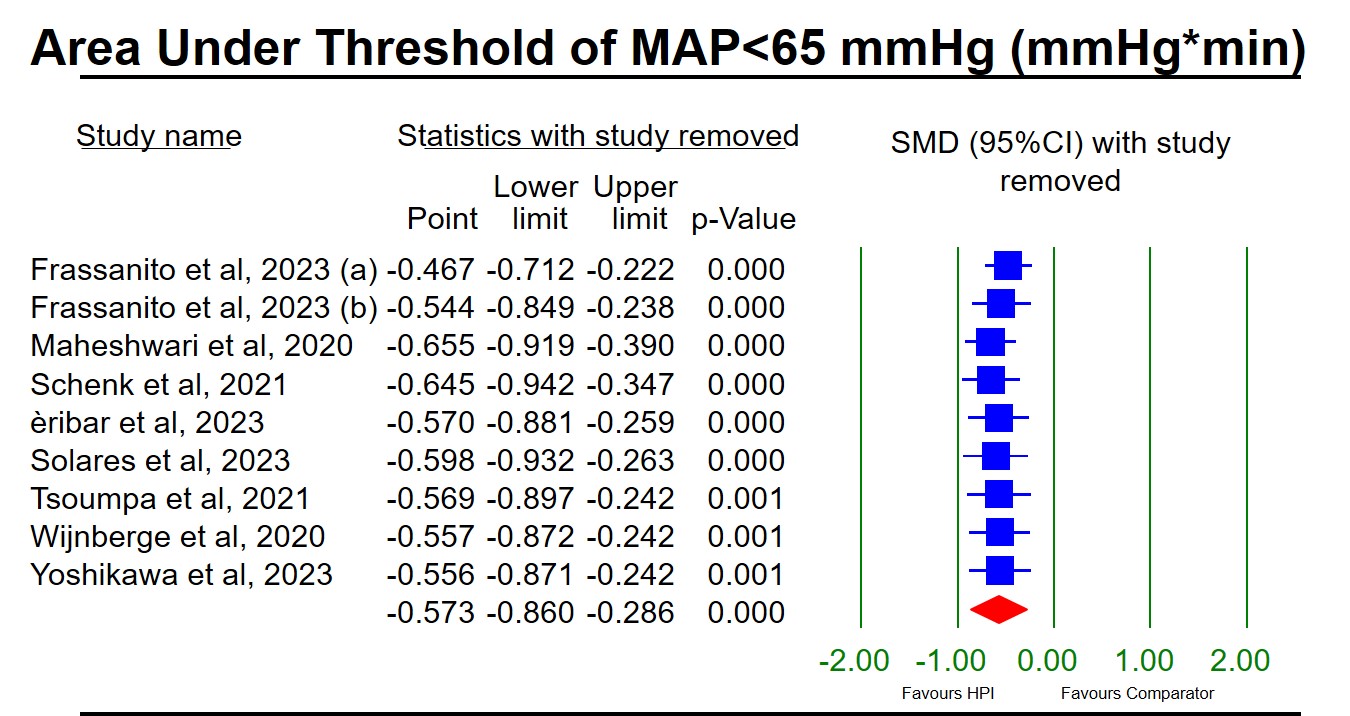


(a): (1)
(b): (2)

Figure S6: Funnel plot for assessing the publication bias of our meta-analysis of the area under the receiver operating curve for non-hypotension prediction index artificial intelligence models for the prediction of intraoperative hypotension. The plot is asymmetric.

**
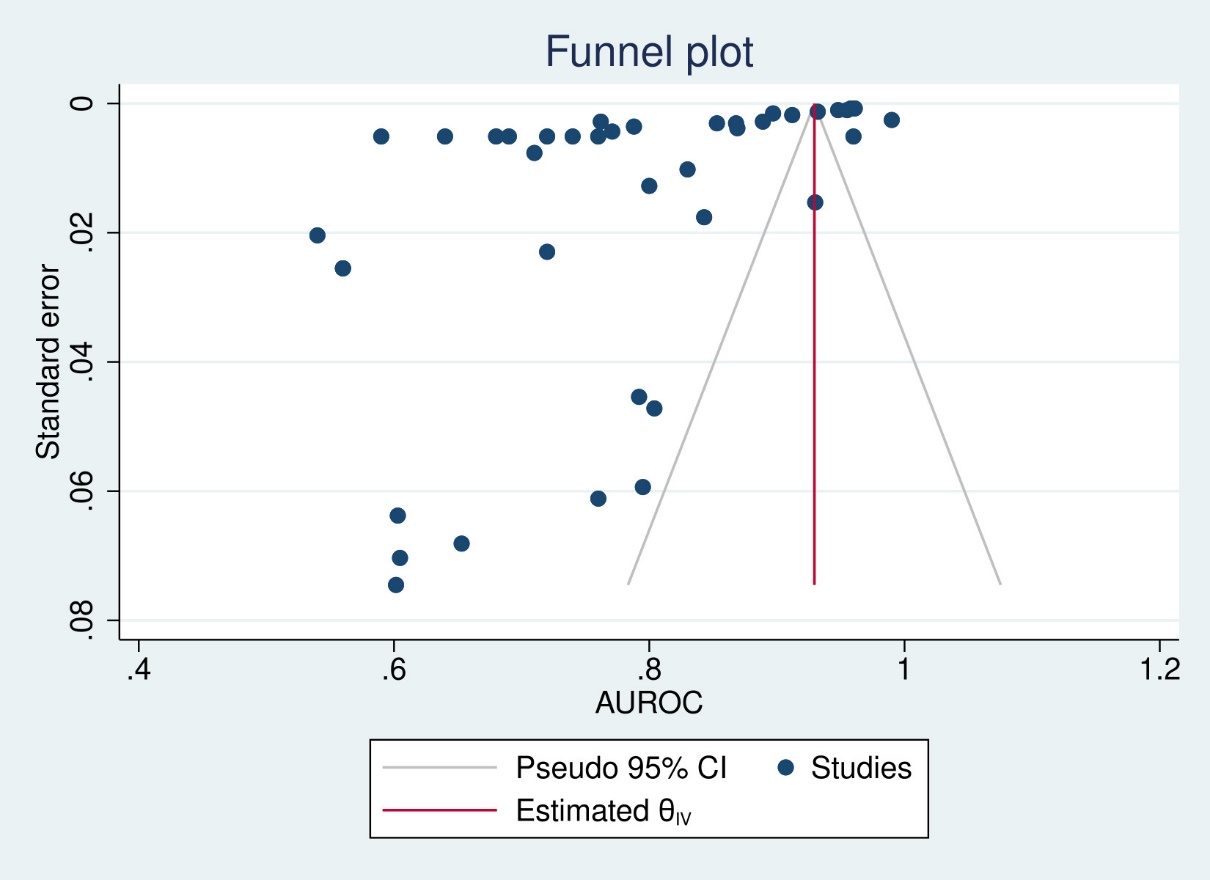
**

Figure S7: Sub-group analysis of the AUROC meta-analysis for non-HPI studies based on the cut-off used to define hypotension.


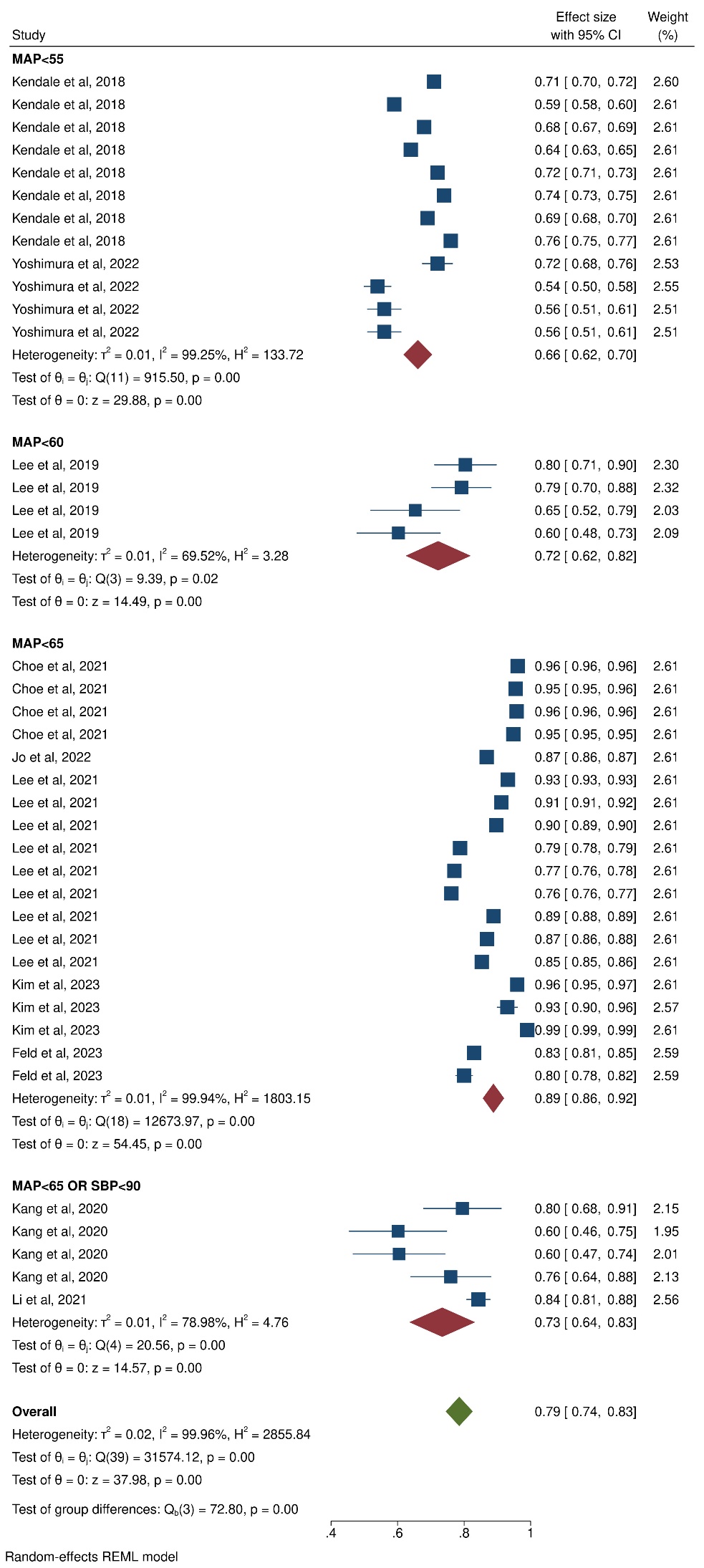


Figure S8: Sub-group analysis of the AUROC meta-analysis for HPI studies 5 minutes prior to intraoperative hypotension based on study quality and risk of bias.


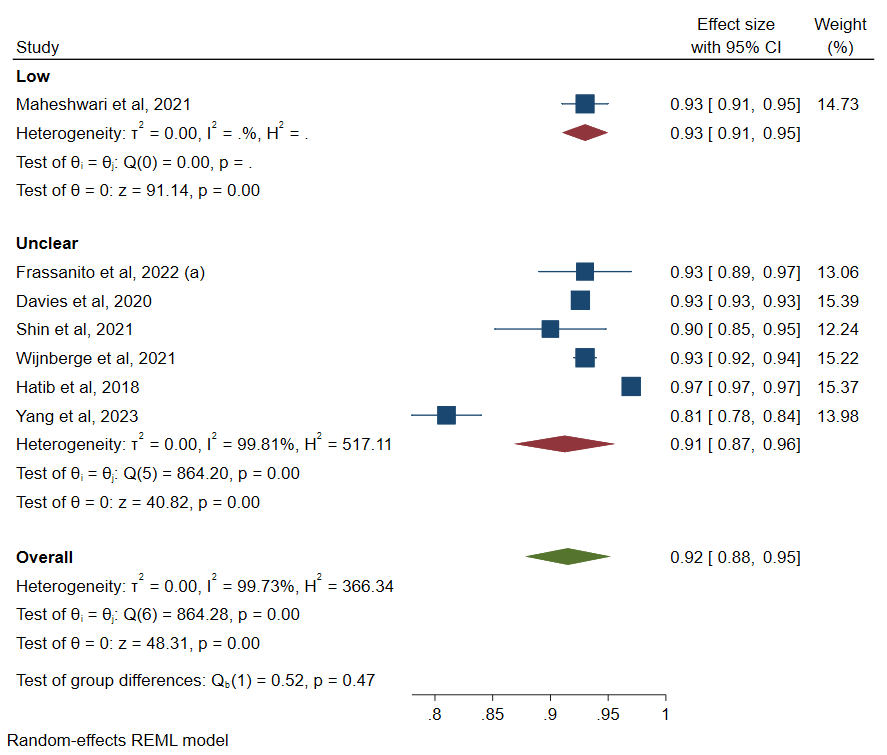


Figure S9: Sub-group analysis of the AUROC meta-analysis for HPI studies 10 minutes prior to intraoperative hypotension based on study quality and risk of bias.


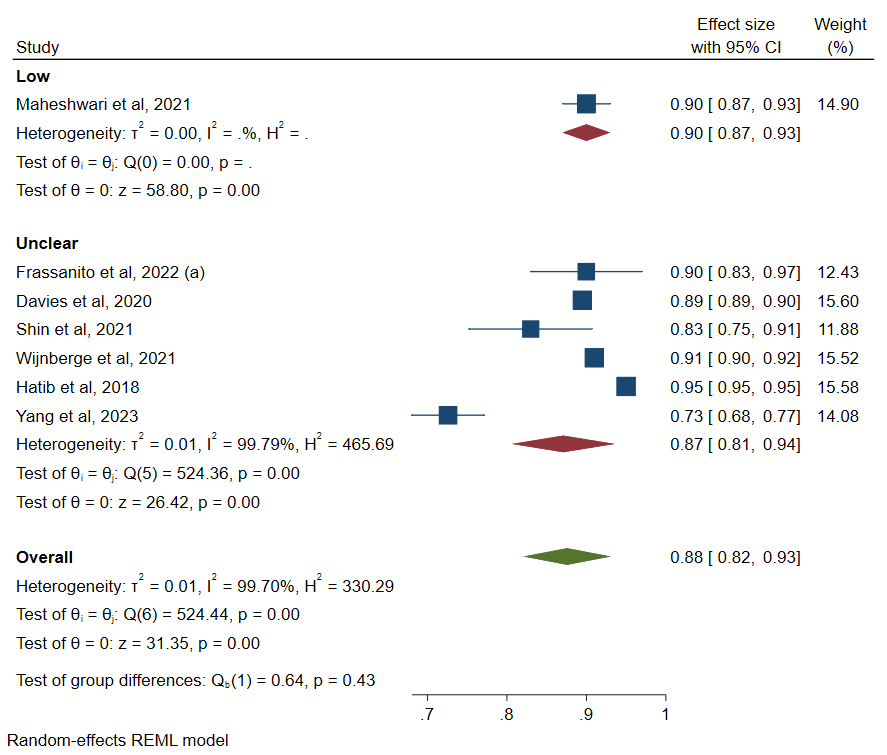


Figure S10: Sub-group analysis of the AUROC meta-analysis for HPI studies 15 minutes prior to intraoperative hypotension based on study quality and risk of bias.


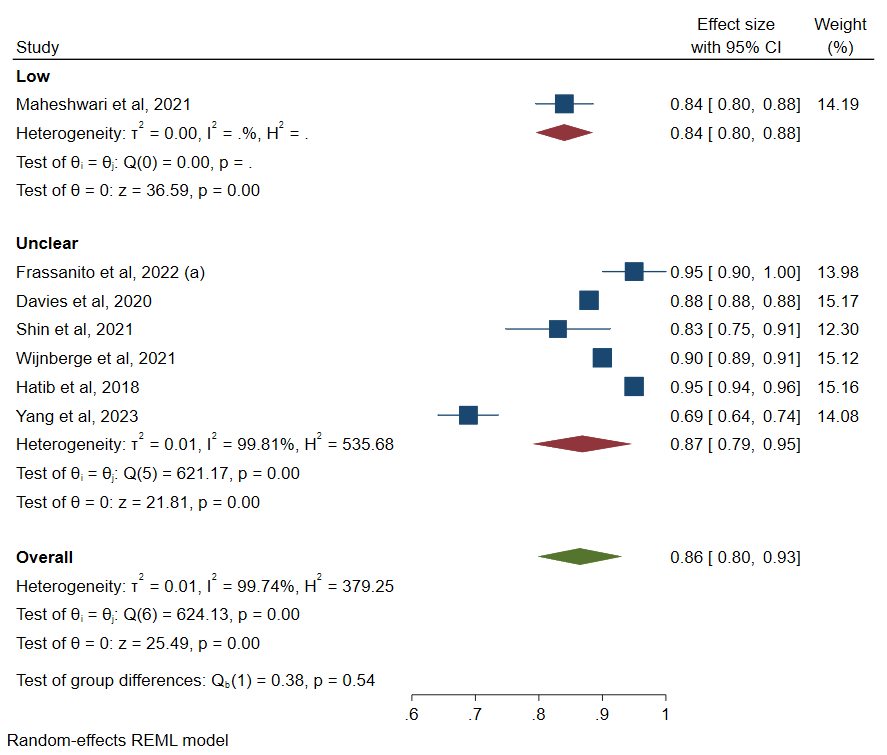


Figure S11: Sub-group analysis of the AUT-MAP<65 meta-analysis for HPI studies based on study quality and risk of bias.


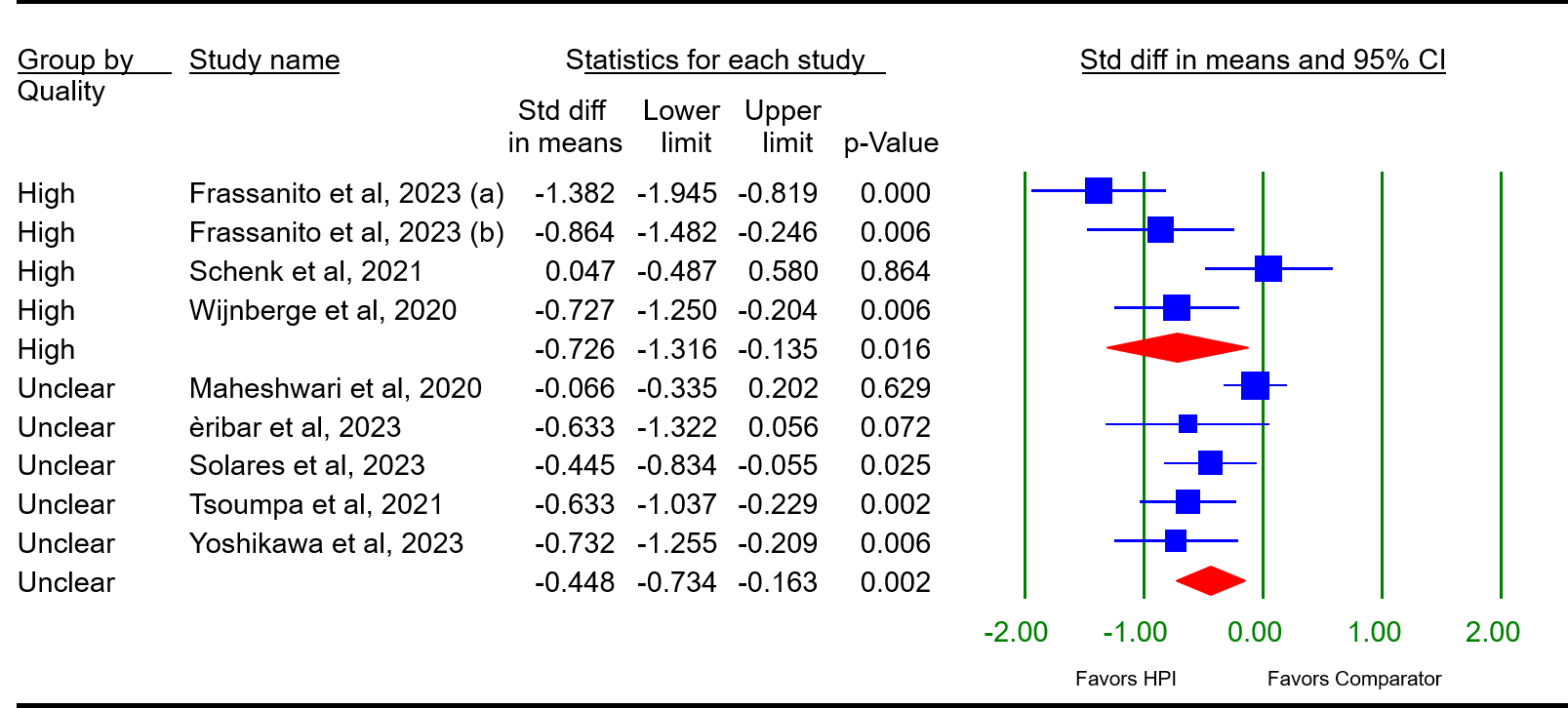


Figure S12: Sub-group analysis of the duration of intraoperative hypotension meta-analysis for HPI studies based on study quality and risk of bias.


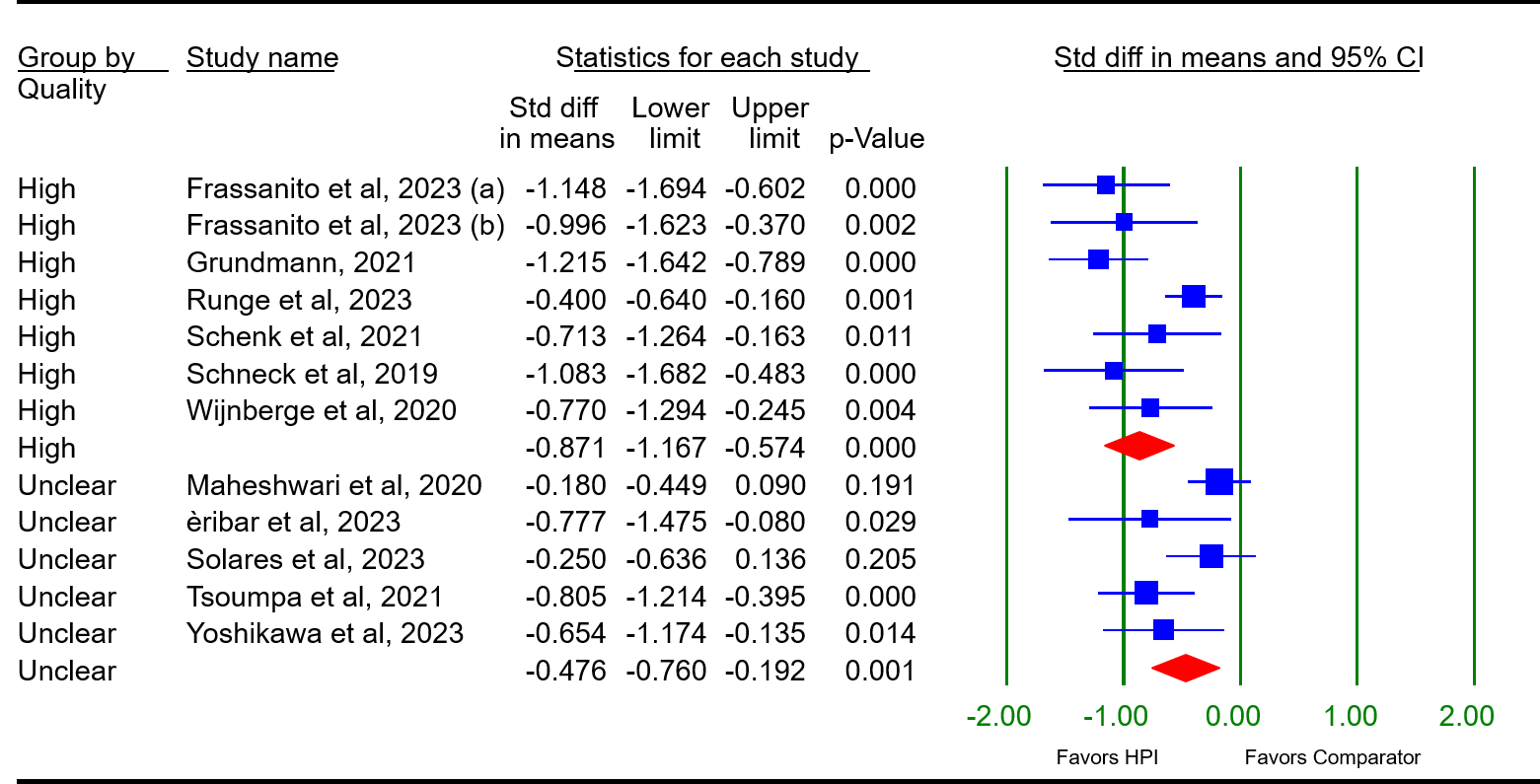


Figure S13: Sub-group analysis of the TWA-MAP<65 meta-analysis for HPI studies based on study quality and risk of bias.


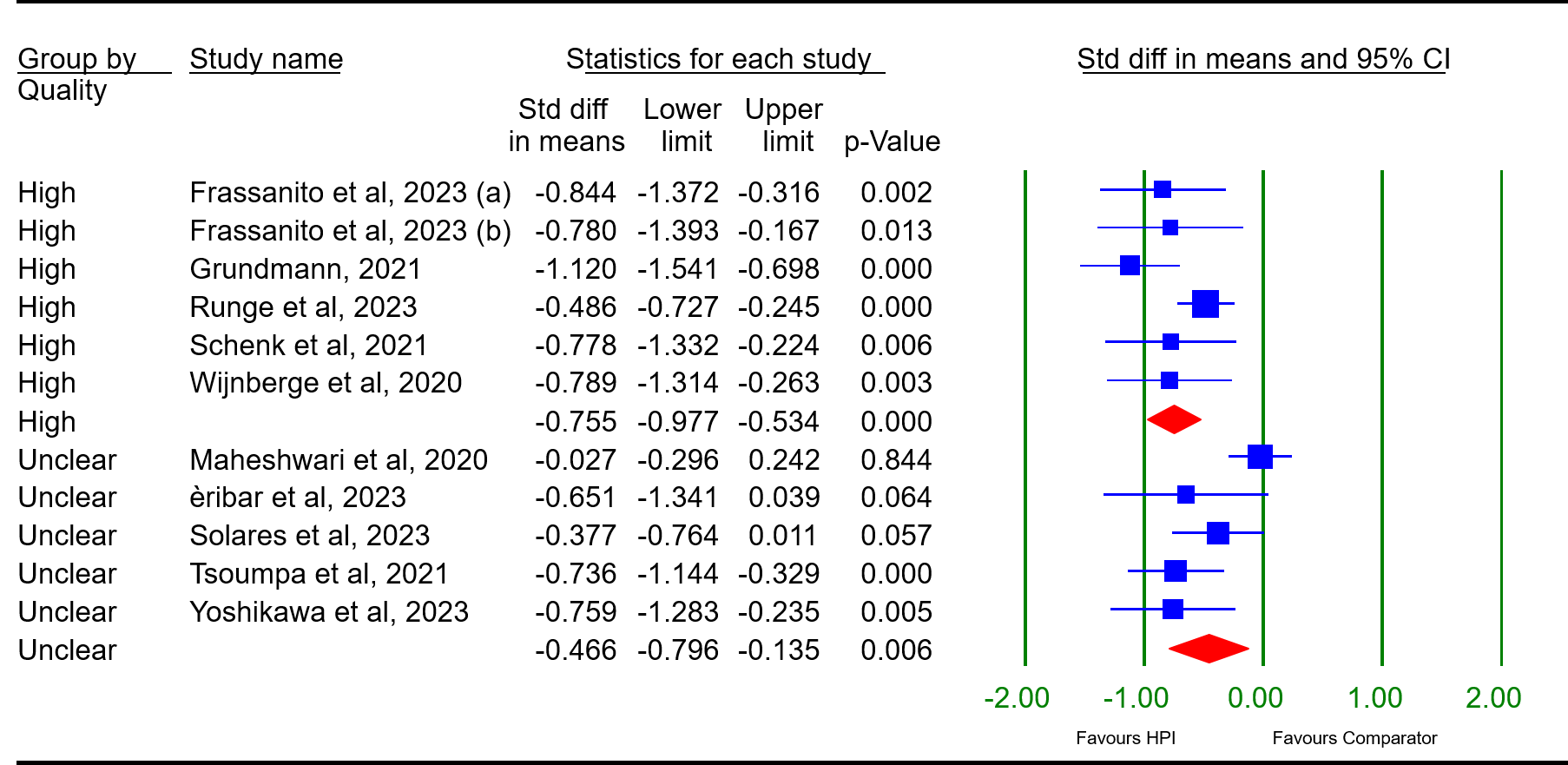


Figure S14: Sub-group analysis of the AUROC meta-analysis for non-HPI studies based on study quality and risk of bias.


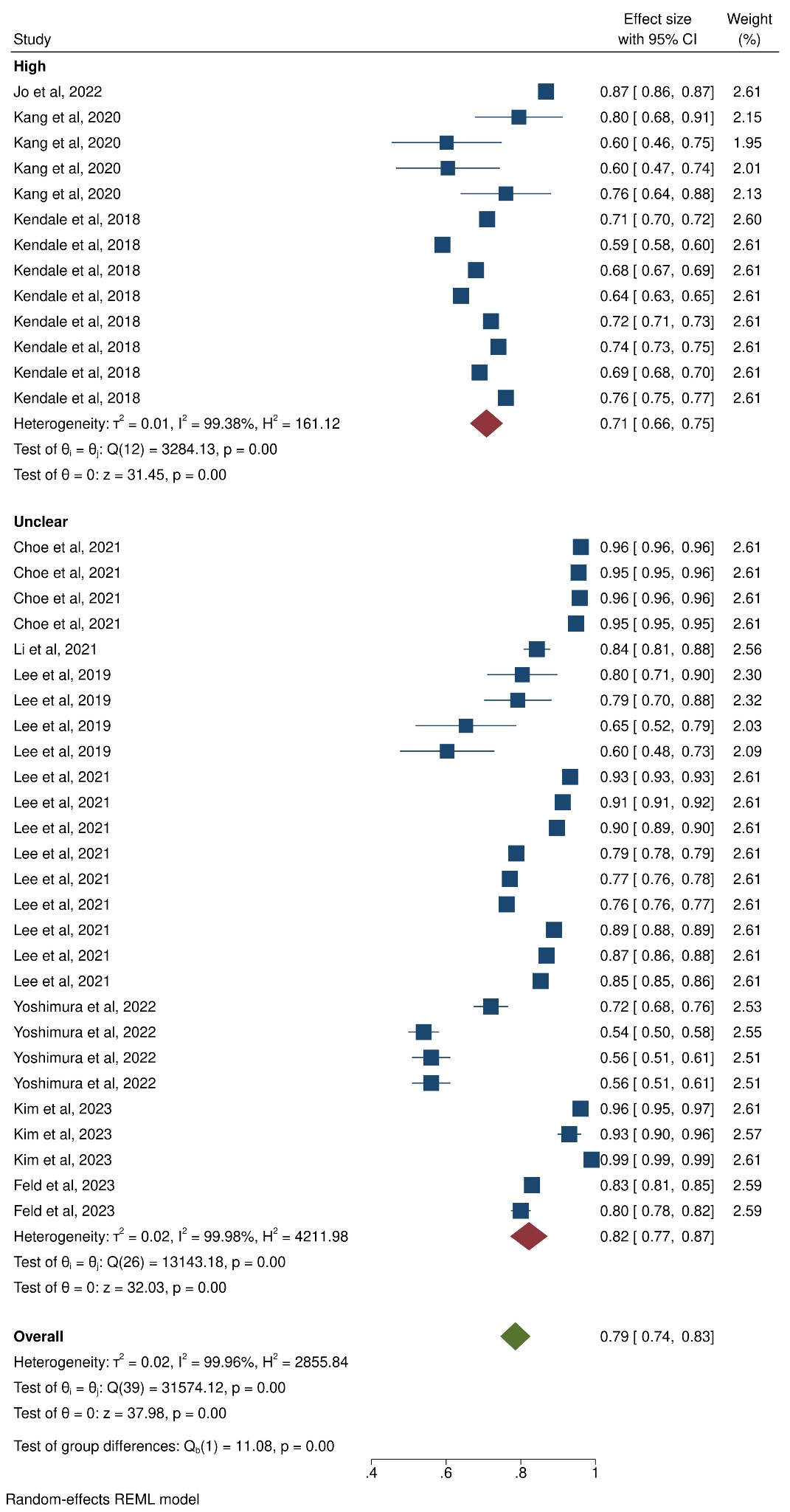


**References:**

1. Frassanito L, Giuri PP, Vassalli F, Piersanti A, Garcia MIM, Sonnino C, et al. Hypotension Prediction Index guided Goal Directed therapy and the amount of Hypotension during Major Gynaecologic Oncologic Surgery: a Randomized Controlled clinical Trial. J Clin Monit Comput. 2023;37(4):1081-93.

2. Frassanito L, Giuri PP, Vassalli F, Piersanti A, Zanfini BA, Catarci S, et al. Noninvasive hypotension Prediction Index versus continuous blood pressure monitoring and intraoperative hypotension. Minerva Anestesiol. 2023;89(6):603-5.
