# Supplementary material for: Predictive ability of hypotension prediction index and machine learning methods in intraoperative hypotension: a systematic review and meta-analysis
Source: J Transl Med. 2024 Aug 5;22:725. doi: 10.1186/s12967-024-05481-4 (PMC11302102; doi:10.1186/s12967-024-05481-4)
Supplement: Supplementary file 3 — Supplementary Material 3 [file 12967_2024_5481_MOESM3_ESM.docx]

| Number of reviewer | query | response | Line and page added with highlight |
| --- | --- | --- | --- |
| 1 | Please define the Timing of the PICOS | Thank you for pointing out this flaw in our manuscript. For the purposes of our study intra-operative hypotension was defined as hypotension occurring after the induction of anesthesia, regardless of the specific timeframe during which it was measured by different studies. | Methods  Page 4  Lines 85-88 |
| 1 | Does the "intraoperative hypotension" mean the interval between the anesthetic induction and the end of resuscitation, or the interval between the beginning and the end of surgery? | Definitions vary across the articles included. A column containing the exact timeframe of IOH was added to Table 2 to clarify the ambiguity of the term. | Tables  Page 22 |
| 1 | It would have been better to include a definition of "low blood pressure" for each study included in the supplement (Was the definition of hypotension consistent across the included studies?) | A column containing the exact cut-off was added to Table 2 to rectify this issue | Tables  Page 22 |
| 1 | The machine learning methods were also used in predicting and preventing hypotension-induced complications, including sepsis (PMID: 36790357) and AKI (PMID: 34321016), this could help to illustrate the significance of the study. | Thanks for your precious comment and introducing useful articles. This point was added to the introduction to illustrate the significance. | Introduction  Page 3  Lines 60-61 |
| 1 | Was there any information on patients with hypertension or cerebrovascular disease in the included studies population, or exclusions? | We are grateful for your suggestion. A column containing the inclusion and exclusion criteria used in each study was added to Tables S1 and S2. | Results  Page 7 line 163  Page 8 line 209  And Additional file 1 |
| 1 | Does the definition of hypotension in this population follow MAP<65mmHg or others? | Cut-offs for hypotension in non-HPI studies are provided in Table 2. In addition, it was pointed out that HPI studies use a MAP<65 mmHg in the discussion. | Tables  Page 22  Discussion  Page 11  Line 291-296 |
| 1 | In Data extraction, it is better to add more information of the models of the included studies, i.e.: the method of missing data handling, feature selection method, final predictors. | Thanks for your valuable comment .The information was added to Table S2 | Page 8 line 209  And Additional file 1 |
| 2 | It is hoped to conduct appropriate subgroup analysis of the result indicators, and list and write tables appropriately. | Thank you for your insightful comment. Further subgroup analyses were conducted based on the cut-off for hypotension. There was not sufficient information to conduct other subgroup analyses. | Results  Page 9  Lines 223-226 |
| 2 | When only high-quality studies were included, whether IOH duration TWA-MAP<65 mmHg and AUT-MAP<65 mmHg were significantly reduced in the high-quality study group using HPI compared to the high-quality study group without HPI. | As you suggested, further subgroup analyses were conducted based on the quality of the included studies to determine the effect of quality on the results we have obtained | Results  Page 9  Lines 233-236  and 239-241 |
| 2 | More suggestions for future research in this area can be added to the discussion, such as more indicators, more reasonable design, etc., to improve the quality of the original study. So that the subsequent meta-analysis can be better | As recommended, more suggestions were added to the limitation section of discussion. | Discussion  Page 12  Lines 309-312 |
